# Native Metabolomics Unveils Suomilide Analogs with Potent Trypsin Inhibitory Activity

**DOI:** 10.1021/acs.jnatprod.5c01380

**Published:** 2026-04-01

**Authors:** Amira Naimi, Christoph Ulbricht, Tung Lam Trinh, Stefan Kehraus, Teresa Marina Dreckmann, Uwe Linne, Kornelia Hardes, Raphael Reher, Martin Baunach

**Affiliations:** † Institute of Pharmaceutical Biology and Biotechnology, Marburg University, Robert-Koch-Str. 4, 35037 Marburg, Germany; ‡ Institute of Pharmaceutical Biology, 9374 University of Bonn, Nussallee 6, 53115 Bonn, Germany; § Department of Chemistry, 9377 Philipps-Universität Marburg, Hans-Meerwein-Straße 4, 35043 Marburg, Germany; ∥ 652666 Fraunhofer Institute for Molecular Biology and Applied Ecology - Branch for Bioresources, Ohlebergsweg 12, 35392 Gießen, Germany

## Abstract

Cyanobacteria are well-known for their ability to produce a diverse array of serine protease inhibitors and thus are a promising source of pharmaceutical lead structures. Here, native metabolomics was employed with trypsin to rapidly screen whole cyanobacterial metabolomes and uncover new potent inhibitors. In combination with subsequent correlative LC-HR-MS/MS-based analysis, this led to the fast discovery of the well-characterized natural product family of suomilides from *Nostoc* sp. KVJ20 as highly potent trypsin inhibitors. Notably, two of the six putative binders were recognized as potential unknown analogs and thus were chosen for isolation and structural elucidation by means of 1D/2D NMR spectroscopy, Marfey’s analysis, and bioinformatic investigations. This resulted in the identification of new suomilides G and H (**1**/**2**), which were isolated together with the known congeners suomilide B (**3**) and D (**4**). We also demonstrated that all four isolated suomilides inhibit trypsin at low nanomolar to high picomolar concentrations in a competitive bioassay, confirming the exceptional potency of this growing compound family as protease inhibitors.

## Graphical abstract

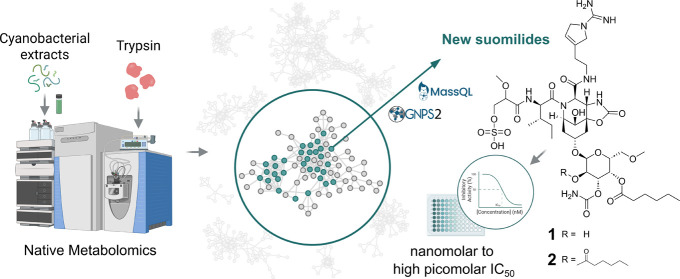

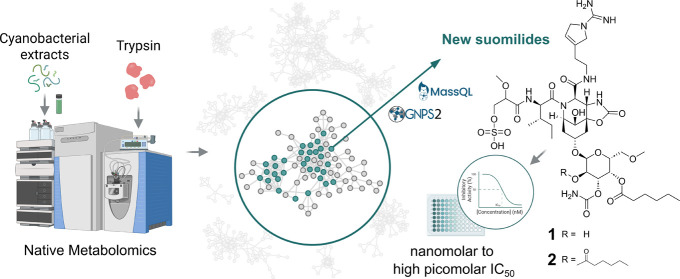

Cyanobacteria are a promising sources of diverse, potent bioactive natural products that are known for their antibacterial, antiviral, antifungal, anti-inflammatory, and anticancer activities.^1–3^ Due to the high diversity of cyanobacterial natural products,^4,5^ we have focused on screening a library of different cyanobacterial extracts with an affinity-selection mass spectrometric approach, native metabolomics^6,7^ that enables us to detect presence/absence of binding for all ionizable metabolites in a complex extract against target proteins of interest. Thus, native metabolomics enables highly effective and targeted analysis of entire metabolomes of various sources in a time-efficient manner.

Among bacteria, cyanobacteria are known to be very prolific producers of serine protease inhibitors belonging to various structural classes, ranging from simple dipeptides to complex peptides, such as aeruginosins,^8^ microviridins,^9,10^ anabaenopeptins,^11^ Ahp-cyclodepsipeptides,^12,13^ and largamides.^14^ Many cyanobacterial strains simultaneously produce different classes of protease inhibitors, which are suggested to serve as feeding deterrents against grazers.^15^ These compounds exhibit selective inhibitory effects against various serine proteases, including chymotrypsin, elastase, trypsin, thrombin, and plasmin, reflecting both ecological defense roles and pharmacological potential.^16^

One of them, trypsin, plays a decisive role when it malfunctions. This protein is linked to the development of many diseases like pancreatitis,^17^ inflammatory lung diseases such as cystic fibrosis and chronic obstructive pulmonary disease,^18^ and the progression of specific cancer types.^19,20^ In addition, the infection process with several RNA-viruses such as SARS-CoV-2 and influenza viruses is also facilitated by trypsin-like serine proteases.^21,22^ Thus, trypsin has been the subject of scientific research for many years as a potential target for therapeutic interventions.^23–27^

To accelerate the search for potent new bioactive natural products targeting trypsin, in this study, we apply native metabolomics for the first time against the serine protease trypsin, more specifically porcine trypsin that is screened against several cyanobacterial metabolomes. By correlating the retention times of the newly observed signals corresponding to trypsin/small-molecule complexes in native metabolomics runs with those in subsequent metabolomics runs, new natural products can be rapidly dereplicated and prioritized using computational approaches such as GNPS2,^28,29^ SIRIUS,^30^ CANOPUS,^31^ and MassQL.^32^ Conceptually related affinity-selection mass spectrometry (AS-MS) approaches have been developed through the pioneering efforts of multiple laboratories to detect noncovalent protein–ligand interactions and characterize intact complexes in the gas phase.^33–38^ In the context of natural products, AS-MS has been applied primarily to purified compounds or fractions to assess target binding.^39,40^ Building on these advances, native metabolomics extends native MS to metabolome-wide screening directly from complex extracts and enables the direct linkage of protein-binding events for all ionizable metabolites in an extract with LC-MS/MS-based metabolomics data, thereby facilitating targeted isolation and structural characterization of bioactive metabolites.

Here, native metabolomics analysis of eight cyanobacterial extracts revealed *Nostoc* sp. KVJ20 ^41^ as the most promising source for trypsin binders, including putatively new suomilide analogs that were targeted for isolation and structure elucidation via AI-assisted 1D/2D NMR spectroscopy, Marfey’s analysis,^42^ and biosynthetic considerations. We elucidate the structure of the new suomilides G and H (**1**/**2**) and confirmed the bioactivity of all isolated suomilides in an orthogonal bioassay as highly potent trypsin inhibitors acting in the low nanomolar to high picomolar range.

## Results and Discussion

### Native Metabolomics-Guided Screening

Cyanobacterial extracts are first separated using reversed phase microflow ultrahigh performance liquid chromatography (μ-flow UHPLC). The eluate is conditioned to near-physiological, nondenaturing conditions by adding NH_4_Ac buffer via a makeup-flow of using an additional isocratic pump module. Orthogonally to this, a continuous stream of the trypsin is infused via a syringe pump downstream of the separation-column and the isocratic pump, so that highly concentrated eluting small molecules could interact with the protein prior to electrospray, allowing intact protein–ligand complexes to be detected for in theory all ionizable small molecules in a complex extract by native mass spectrometry (Figure 1). A second, complementary measurement to the native metabolomics run is performed as a conventional metabolomics run without addition of protein, but also with the makeup flow to retain the retention times. This results in LC-MS/MS data of all binders that can be used for downstream analysis such as MS1-level and MS/MS-based dereplication and prioritization.

**Figure 1:**
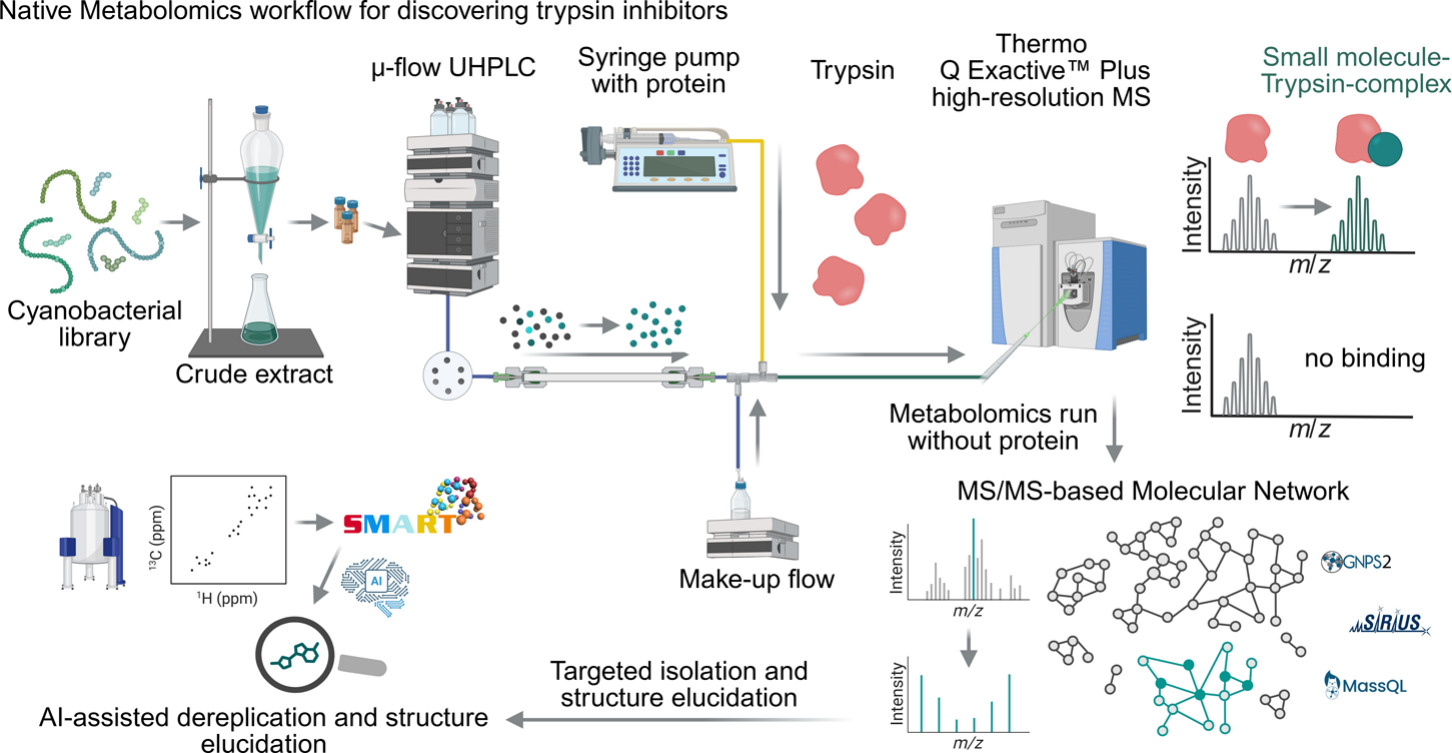
Native metabolomics workflow for discovering trypsin inhibitors. Cyanobacterial extracts are initially separated using μ-flow UHPLC. After the column, the eluent’s pH is adjusted with an excess of NH_4_Ac solution via a makeup pump to simulate native-like conditions. Simultaneously, trypsin is continuously infused in NH_4_Ac solution via a syringe pump. The resulting protein or protein-small molecule complexes are analyzed using native MS conditions. Concurrently, a separate metabolomics run is conducted, featuring UHPLC-HR-MS/MS acquisition without the infusion of protein to enable correlation of retention times and MS1 level data of the two runs. Created in BioRender. Reher, R. (2026) https://BioRender.com/2jhyps4.

To test the suitability of the native metabolomics approach for porcine trypsin and to optimize the MS parameters, we first measured trypsin from porcine pancreas in direct native MS experiments (Figure 2A). Native activated porcine trypsin consists of a single chain polypeptide of 223 amino acid residues, that is cross-linked by six disulfide bridges and has a theoretical monoisotopic mass of 23,448.424 Da.^43^ In our native MS experiments we could confirm native activated porcine trypsin with a deconvoluted mass of 23,448.350 Da (Figure S1). Next, to ensure that measured proteins were intactly folded and to check whether binders can be identified in the setting, we incubated trypsin with the known benzamidine-related serine protease inhibitor benzylsulfonyl-d-argininyl-proline-(4-amidinobenzyl), short BAPA as a positive control.^44^ In this experiment we clearly could detect a novel signal with a mass shift corresponding to BAPA binding (Figure 2B,C). Consequently, we are able to assess binding of trypsin ligands to native porcine trypsin under the applied measurement conditions.

**Figure 2:**
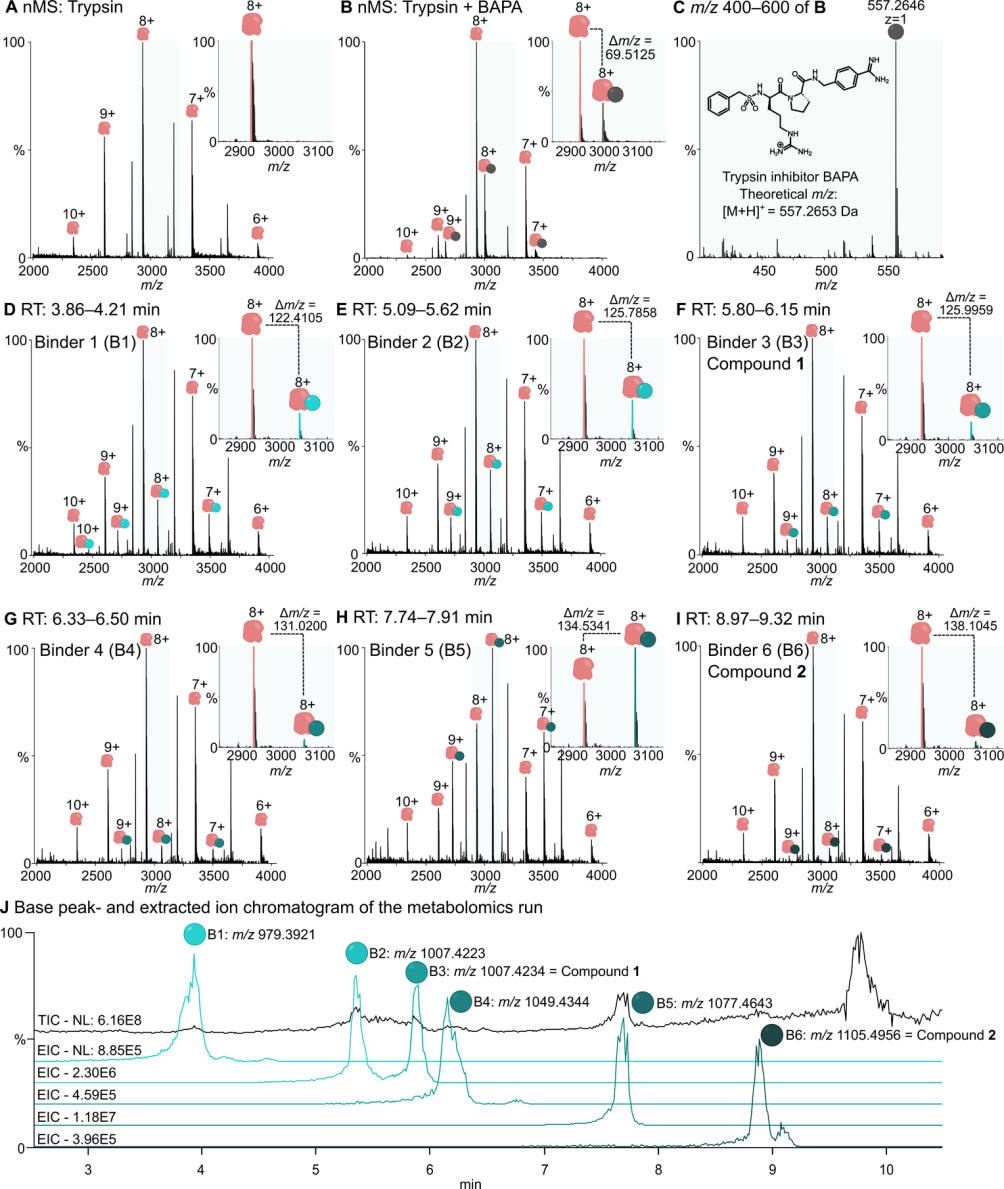
Native metabolomics analysis of trypsin versus *Nostoc* sp. KVJ20 extract. (A) Direct infusion native mass spectrometry measurement of trypsin. (B) Direct infusion native mass spectrometry measurements of protein-inhibitor interactions. Positive control with the confirmed benzamidine-based trypsin inhibitor BAPA^44^ (*m*/*z* 557.2646). The gray circle indicates mass shifts corresponding to the trypsin-BAPA complex. (C) Low *m*/*z* range to detect the nonbinding portion of BAPA. (D) Native metabolomics run at retention time (RT) 3.86–4.21 min. Mass shift of binder 1 (Δ*m*/*z* 979.4765) binding to trypsin in light turquoise. (E) Native metabolomics run at RT 5.09–5.62 min. Mass shift of binder 2 (Δ*m*/*z* 1007.7051) in turquoise. (F) Native metabolomics run at RT 5.80–6.15 min. Mass shift of isomeric binder 3 (Δ*m*/*z* 1007.9610, compound **1**) in teal. (G) Native metabolomics run at RT 6.33–6.50 min. Mass shift of binder 4 (Δ*m*/*z* 1048.4770) in dark teal. (H) Native metabolomics run at RT 7.74–7.91 min. Mass shift of binder 5 (Δ*m*/*z* 1077.8262) in petrol green. (I) Native metabolomics run at RT 8.97–9.32 min. Mass shift of binder 6 (Δ*m*/*z* 1106.0389, compound **2**) in dark petrol green. (J) Base peak chromatogram (BPC) of the subsequent metabolomics run of the extract of *Nostoc* sp. KVJ20. Extracted ion chromatograms of the *m*/*z* 979.3921, *m*/*z* 1007.4223, *m*/*z* 1007.4234 (compound **1**), *m*/*z* 1049.4344, *m*/*z* 1077.4643, *m*/*z* 1105.4956 (compound **2**). For correlation of native metabolomics and metabolomics runs, please refer to Table S1.

With a functional screening assay at hand, a total of eight cyanobacterial strains were screened using native metabolomics as described above, of which the extract of *Nostoc* sp. KVJ20 revealed several new signals that were identified as potential binders (Figure 2D–I; Table S1).

Six putatively new trypsin binders (B1–B6) with averaged mass shifts (Δ*m*/*z* of B1 = 979.4765, Δ*m*/*z* of B2 = 1007.7051, Δ*m*/*z* of B3 = 1007.9610, Δ*m*/*z* of B4 = 1048.4770, Δ*m*/*z* of B5 = 1077.8262, and Δ*m*/*z* of B6 = 1106.0389, see Figure 2D–I; Table S1) were observed in the native metabolomics run with *Nostoc* sp. KVJ20 extracts. In the subsequent metabolomics runs, retention times and MS1 data for all six binders could be correlated with high confidence to the detected binders of the native metabolomics run (Δ*m*/*z* between native metabolomics and metabolomics runs were <1 Da for all putative binders (Figure 2J; Table S1)). After optimized metabolomics conditions, preprocessing of the subsequent LC-MS/MS data in MZmine4 ^45^ and analyzing the output in SIRIUS and GNPS2, we discovered that all of the new potential binders were linked by a particular MS2 fragmentation pattern, especially in the low *m*/*z* range. It turned out that all the binders belonged to one family of compounds (Figure 3), possibly to the family of suomilides as judged by SIRIUS/CANOPUS analysis (Figure S2) and comparison of high resolution MS1 and MS/MS data of suomilide D with literature references.^41^ We then applied MassQL to streamline the targeted isolation. Thus, we were able to identify the molecular family in one cluster, harboring all the potential binders (Figures 3 and S3). While compound **1** (*m*/*z* 1007.4249) likely is an isomer of the known suomilide D,^41^ compound **2** (*m*/*z* 1105.4995) did not match any previously reported suomilide. Thus, both their chemical structures had not been reported yet. We therefore targeted compounds **1** and **2**, together with other putatively known suomilides, for isolation and structure elucidation in this study.

**Figure 3:**
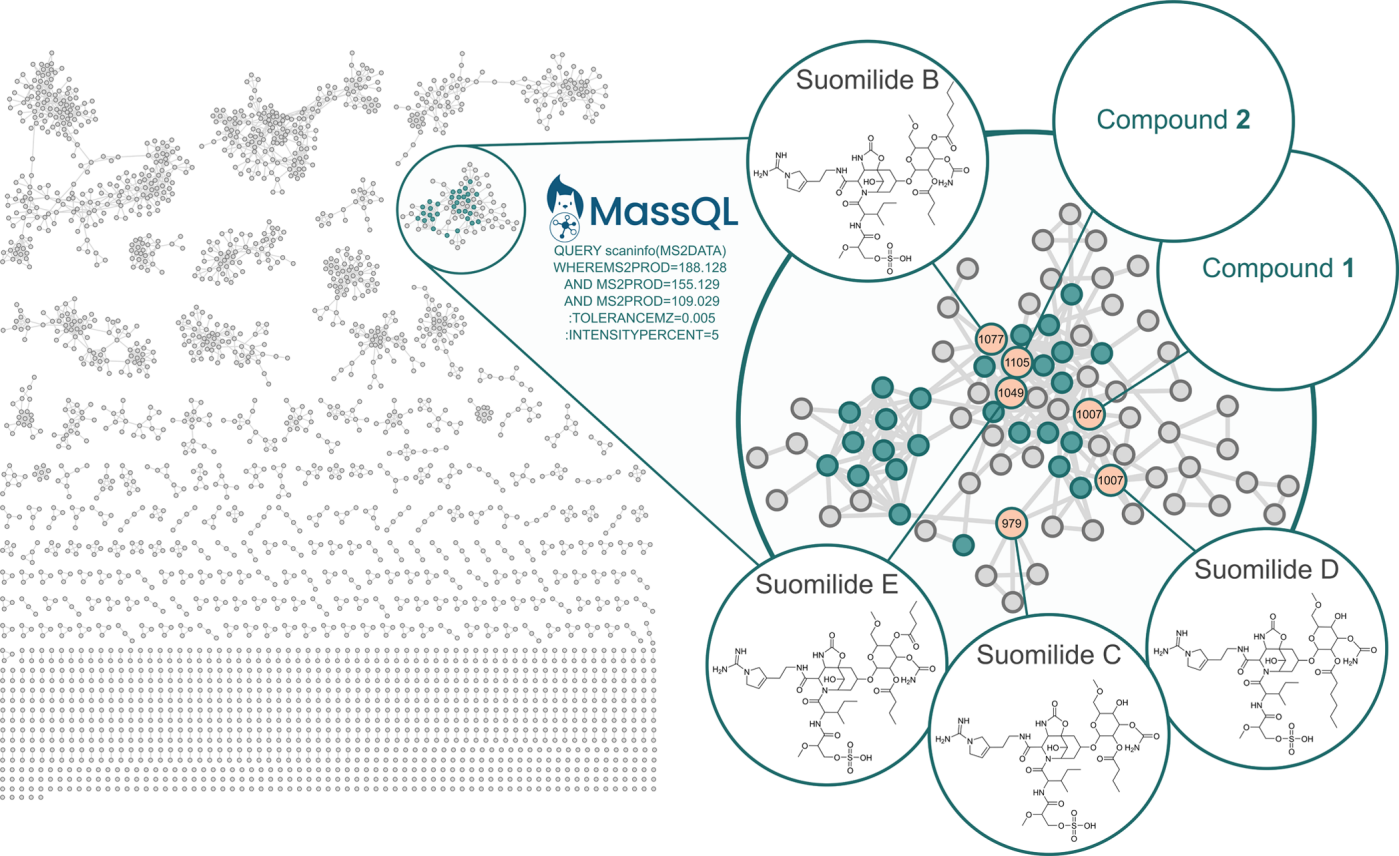
Feature-based molecular network of a LC-HR-MS/MS data set of *Nostoc* sp. KVJ20 with tailored MassQL queries for the native metabolomics derived trypsin binders. We chose the MS2 fragments that all the binders had in common, with *m*/*z* values of 188.128, 155.129, and 109.029, nodes highlighted in cyan with the binders, detected via native metabolomics highlighted in orange. The *m*/*z* tolerance was set at 0.005 Da and the minimum intensity percentage of 5% compared to the MS2 base peak. The structures of compounds **1** and **2** were unknown but suspected to be suomilide congeners due to their clustering with known suomilides, shared MS/MS fragments, a characteristic sulfate loss during LC-MS measurement (Figure S3), and trypsin binding in native metabolomics.

### Isolation and Structure Elucidation

Compound **1** (Figure 4) was isolated as a colorless film. Based on HR-ESI-MS measurements and NMR spectral data (Tables 1 and S2) its molecular formula was determined to be C_41_H_66_N_8_O_19_S. The ^1^H NMR spectrum showed resonances between δ 3.6 and 5.3 ppm characteristic for a sugar residue and α-CH groups indicating compound **1** to be a glycopeptide (Figure S4). A detailed analysis of the NMR spectral and MS data in combination with a comprehensive database search showed compound **1** to have high structural similarities to the suomilides in general, and especially to suomilide D, having the same molecular formula.^41^ SMART 2.1 ^46^ and DeepSAT^47^ analyses (Figure S5) as well as in-depth manual 1D/2D NMR data interpretation (^1^H, ^13^C, ^1^H–^1^H COSY, ^1^H–^13^C HSQC, ^1^H–^1^ ^3^C HMBC, ^1^H–^1^H ROESY Figures S6–S10 and S20; Table S2) confirmed this assumption and gave evidence for the following substructures: isoleucine (Ile), 1-amidino-3-(2-aminoethyl)-3-pyrroline (Aaep), azabicyclononane (Abn), 2-*O*-methylglyceric acid 3-*O*-sulfate (Mgs), hexanoic acid, and one hexose residue. The sequence of these subunits and finally the planar core structure were established to be the same as in the suomilides based on analyses of ^1^H–^13^C HMBC spectral data.

**Figure 4:**
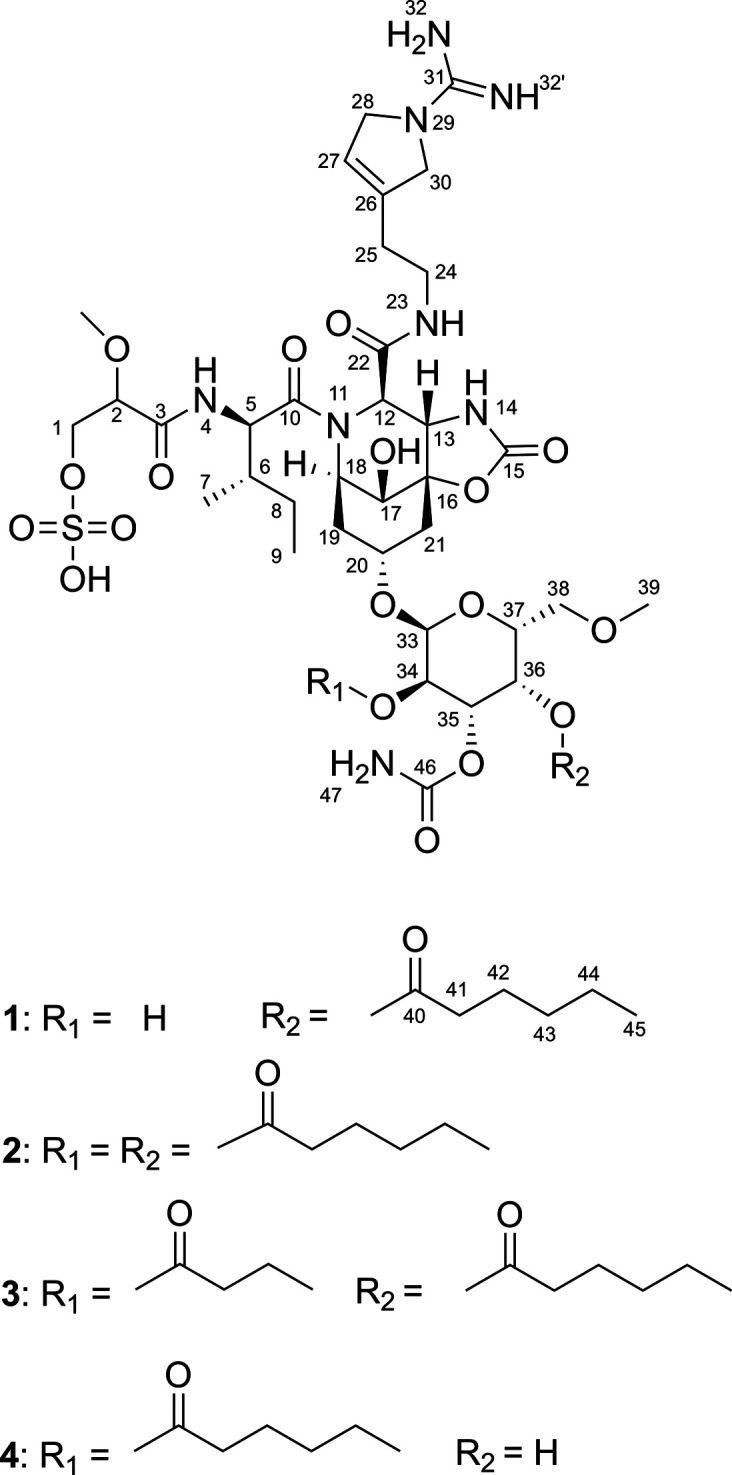
Structures of the new analogs suomilide G (**1**) and suomilide H (**2**) together with the known congeners suomilide B (**3**) and D (**4**).^41^ The yet undefined stereochemistry of **3** and **4** was adapted based on analogy and biosynthetic logic.

**Table 1:** NMR Spectroscopic Data for Compounds **1** and **2** (600 MHz, DMSO-*d*_*6*_)

			compound **1**		compound **2**	
	position		δ_H_ (*J* in Hz)^a^	δ_C_ ^a^	δ_H_ (*J* in Hz)^a^	δ_C_ ^a^
Mgs	1	CH_2_	a: 3.77, m	66.3	a: 3.76, m	66.0
			b: 3.95, m		b: 3.93, m	
	2	CH	3.96, m	80.1	3.93, m	80.0
		OCH_3_	3.31, s	57.4	3.30, s	57.3
	3	C		169.8		n.d.
*allo*-Ile	4	NH	8.02, brd, 7.0		7.91, brd, 6.7	
	5	CH	4.62, t, 7.0	52.8	4.61, brt, 6.7	52.9
	6	CH	1.74, m	36.5	1.72, m	36.5
	7	CH_3_	0.87, d, 7.5	14.5	0.88, d, 7.5	14.2
	8	CH_2_	a: 1.18, m	25.6	a: 1.19, m	25.5
			b: 1.30, m		b: 1.30, m	
	9	CH_3_	0.90, t, 7.4	11.7	0.90, t, 7.5	11.7
	10	C		171.6		n.d.
Abn	11	N				
	12	CH	4.58, d, 2.0	56.9	4.53, d, 1.9	56.6
	13	CH	4.21, brs	58.4	4.23, brs	58.0
	14	NH	8.08, s		8.01, brs	
	15	C		156.7		n.d.
	16	C		80.6		n.d.
	17	CH	3.71, brs	65.7	3.72, brs	65.4
		OH	6.03, brs		6.05, brs	
	18	CH	4.28, brs	53.5	4.25, brs	53.2
	19	CH_2_	a: 1.80, m	28.9	a: 1.75, m	n.d.
			b: 2.17, m		b: 2.15, m	
	20	CH	3.76, m	69.7	3.75, m	69.3
	21	CH_2_	a: 1.95, m	34.6	a: 1.97, m	33.6
			b: 2.30, m		b: 2.30, m	
Aaep	22	C		168.8		n.d.
	23	NH	7.80, brs		7.63, brs	
	24	CH_2_	3.20, m	37.2	3.19, m	36.5
	25	CH_2_	2.29, m	27.8	2.28, m	27.9
	26	C		136.0		n.d.
	27	CH	5.64, s	118.9	5.63, s	118.8
	28	CH_2_	4.12, m	54.2	4.11, m	54.2
	29	N				
	30	CH_2_	4.12, m	55.4	4.12, m	55.3
guanidine	31	C		154.3		n.d.
	32/32′	NH/NH_2_	7.30, brs		7.34, brs	
					7.41, brs	
Gal	33	CH	4.75, d, 3.9	98.3	4.97, d, 3.7	94.3
	34	CH	3.68, m	65.9	4.84, dd, 3.7, 10.9	67.8
		OH	4.92, brs			
	35	CH	4.72, dd, 3.5, 10.8	69.8	5.02, dd, 3.5, 10.9	66.4
	36	CH	5.27, dd, 1.1, 3.5	68.6	5.34, d, 3.5	68.2
	37	CH	4.02, brt, 6.5	67.0	4.14, t, 6.6	66.8
	38	CH_2_	a: 3.22, m	70.2	a: 3.26, m	69.8
			b: 3.29, m		b: 3.34, m	
	39	OCH_3_	3.19, s	58.5	3.21, s	58.5
HA-1^b^	40	C		172.0		n.d.
	41	CH_2_	2.30, m	33.4	2.31, m	33.2
	42	CH_2_	1.54, m	24.2	1.52, m	24.1
	43	CH_2_	1.30, m	30.6	1.27, m	30.5
	44	CH_2_	1.27, m	21.7	1.27, m	21.7
	45	CH_3_	0.85, t	13.8	0.85, t	13.6
	46	C		156.1		
	47	NH_2_	6.40, brs		6.53, brs	
HA-2^b^	48	C				171.9
	49	CH_2_			2.36, m	33.1
	50	CH_2_			1.54, m	24.1
	51	CH_2_			1.27, m	30.5
	52	CH_2_			1.28, m	21.7
	53	CH_3_			0.86, t	13.7

a Assignments are based on extensive 1D and 2D NMR measurements (HMBC, HSQC, COSY).

b HA-1 and HA-2 may be interchanged for compound **2**; n.d. not detected.

However, a detailed comparison of the ^1^H and ^13^C NMR data of compound **1** and suomilide D showed slight deviations in the chemical shifts of the sugar moiety, especially for the chemical shifts of CH-34 and CH-36, indicating differences in the substitution pattern. An HMBC correlation of H-36 (δ_H_ 5.27) to C-40 (δ_c_ 172.0) evidenced the connection of the hexanoic acid residue to C-36 of compound **1**. In addition, a ^1^H–^1^H COSY correlation of the hydroxy group (δ_H_ 4.92) and H-34 (δ_H_ 3.64) as well as an upfield shift of H-34 showed C-34 to have no substituent. Thus, compound **1** and suomilide D are regioisomers differing only in the place of substitution for the hexanoic acid. To determine the sugar component, ^1^H–^1^H coupling constants were analyzed. The hexose moiety was identified as α-galactose due to characteristic coupling constants of H-33 to H-34 (*J*_33,34_ = 3.9 Hz), H-34 to H-35 (*J*_34,35_ = 10.8 Hz), H-35 to H-36 (*J*_35,36_ = 3.5 Hz) and H-36 to H-37 (*J*_35,36_ = 1.1 Hz), respectively. Intensive ^1^H–^1^H ROESY correlations between H-36 and both H-35 and H-37 and a missing correlation between H-34 and H-36 supported this result (Figures S10 and S20; Table S2). The determination of the absolute configuration of galactose after acidic hydrolysis failed due to the limited amount of compound **1**. The isoleucine residue was shown to be d-*allo*-isoleucine using the advanced Marfey’s method after hydrolysis of **1** with 6 m HCl. The absolute configuration of carbon C-2 and of the azabicyclononane moiety remain unresolved, however, due to biosynthetic logic compound **1** most probably has the same configuration as proven by the chemical synthesis of Carreira et al.^48^

Compound **2** could be isolated as a minor metabolite. HR-ESI-MS analysis revealed a molecular formula of C_47_H_76_N_8_O_20_S. From ^1^H, ^13^C and 2D NMR data it became apparent that compound **2** showed high similarity to **1** (Figures S11–S15). A mass difference of + *m*/*z* 98 gave a first hint for the presence of an additional hexanoic acid residue in compound **2**, a fact that could be evidenced by interpretation of the NMR spectral data. The connection of both hexanoic acid residues to C-34 and C-36 of the galactose moiety could not be proven by HMBC correlations due to the limited amount, however, the downfield chemical shift of H-34 (δ_H_ 4.84) evidenced the connection of the second hexanoic acid residue to C-34, completing the planar structure of **2**. The absolute configurations of **2** are supposed to be the same as in **1** due to biosynthetic logic. In accordance with recent suomilide nomenclature^41,49^ **1** and **2** were named suomilide G and H, respectively. The known congeners suomilide B (**3**) and D (**4**) were confirmed based on comparison of ^1^H and ^13^C NMR data (Figures S16–S19) with published data. It should be noted that **1** and **4** theoretically could result from partial hydrolysis of the hexanoate esters from the galactose moiety of **2**. However, compound **2** was highly stable during the course of isolation and also no signs of interconversion were detectable in the corresponding NMR spectra.

### Biosynthetic Proposal

Based on the published gene cluster of *Nostoc* sp. KVJ20 for banyaside and suomilide like molecules (*bsl*)^41^ we propose a biosynthetic scheme for the production of suomilide G (**1**) and H (**2**) (Figure 5). First, the suomilide backbone is biosynthesized by the two bimodular NRPSs BslA and BslJ. The assembly starts with the loading of a glycerate moiety to the first PCP domain by the FkbH-like domain of the loading module, which in analogy to the biosynthesis of other glycerate-derived natural products is likely derived from 1,3-bisphosphoglycerate (1,3-BPG)^50^ and subsequently is methylated and sulfonated by the module’s *O*-methyltransferase (OMT) and sulfotransferase (ST) domains. The second module of BslA incorporates isoleucine, which is predicted to be epimerized by the downstream epimerization domain. Recruitment of l-Ile would thus result in d-*allo*-Ile, which would fit well with the results of the amino acid analysis via advanced Marfey’s method and also fits to the configuration of isoleucine in suomilide A (originally reported as suomilide)^51^ as reported from *Nodularia sphaerocarpa* UHCC 0038 (Figure S21). However, in the structure of varlaxins, which closely resemble suomilides core structure d-Ile is present (Figure S21) due to the ability of VarB’s A-domain to activate l-*allo*-Ile, which subsequently gets epimerized to d-Ile.^52^ In consequence, we analyzed the Stachelhaus codes of the biosynthetic machinery of various related protease inhibitors like suomilide A,^26^ aeruginosin 98 A–C,^53^ pseudospumigin A–F,^54^ or varlaxin 1046A and 1022A.^52^ Notably, all A-domains share the Stachelhaus code DAFFLGVTFK, which results in the incorporation of l-Ile or d-*allo*-Ile, depending on the presence of a downstream epimerization domain except of the A domain from varlaxin biosynthesis, which shares the Stachelhaus code DALWMGGVFK with other l-*allo*-Ile-recruiting A domains (Figure S21; Table S3), thereby corroborating the result of Marfey’s analysis. Next, the first module of BslJ recruits the intriguing Abn moiety, whose complex biosynthesis remains to be elucidated. Finally, the terminal off-loading module of BslJ triggers release of the peptide from the enzyme complex by amide bond formation between the PCP-bound carboxyl group of Abn and the primary amine of Aaep in analogy to the off-loading mechanism that has been proposed for the biosynthesis of Aeruginoside 126A and 126B.^53^ The biosynthesis of Aaep (sometimes also referred to as Aeap – 1-Amino-2-(*N*-amidino-3-Δ3-pyrrolinyl)­ethane) was recently elucidated by Abe and co-workers, who could show that the BslB and BslD homologues Aer3 and AerC catalyze pyrroline ring formation via *N*-prenylation of agmatine (Agma) followed by oxidative carbocyclization.^55^ After release, the tetrapeptide is further decorated by tailoring reactions including glycosylation by the putative glycosyltransferase BslX and acylation of the hexose moiety by the putative membrane bound *O*-acyl transferase (MBOAT) BslP.

**Figure 5:**
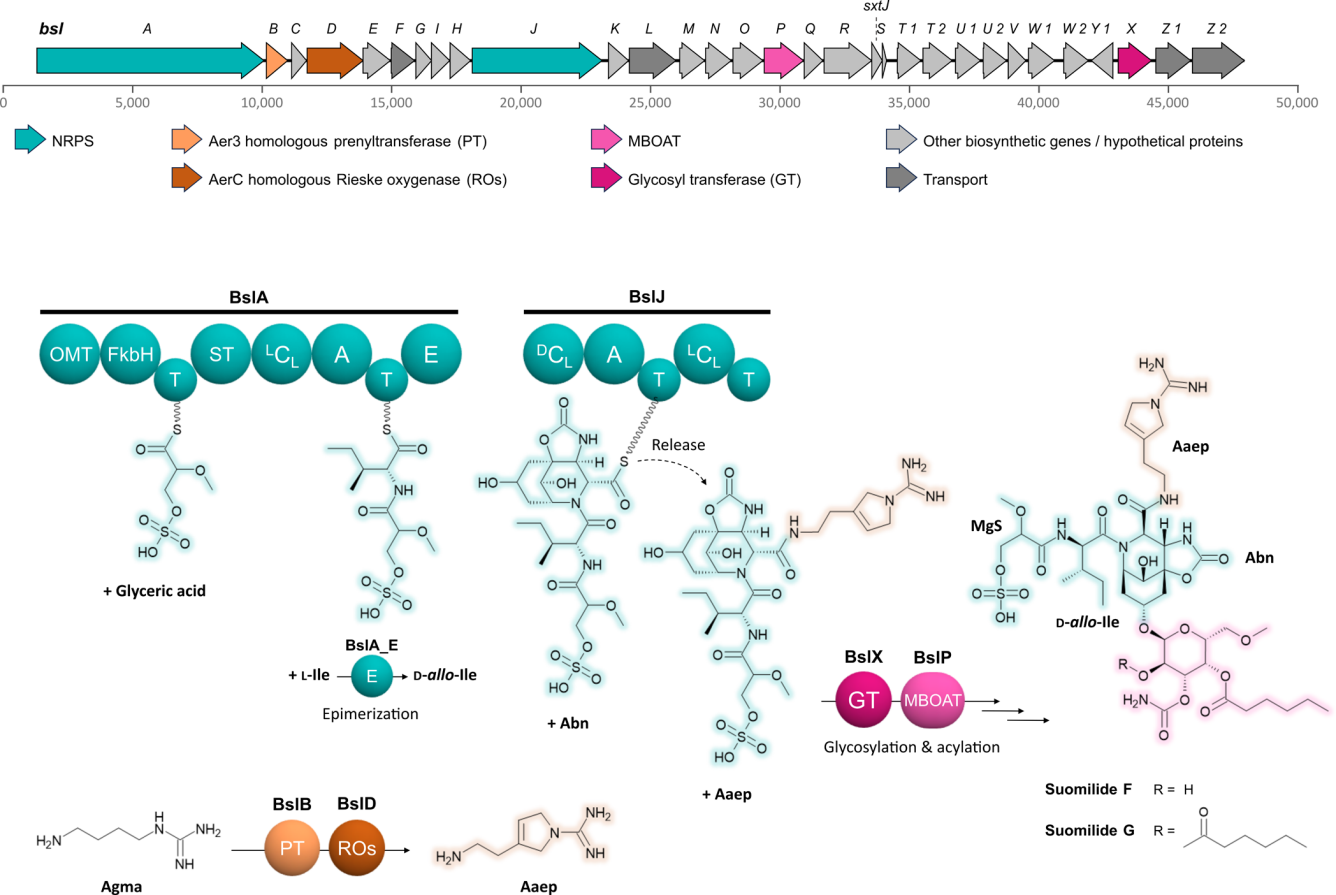
Biosynthetic gene cluster of *Nostoc* sp. KVJ20 for banyaside and suomilide like molecules (*bsl*)^41^ as well as the proposed pathway for the biosynthesis of **1** and **2**. OMT = *O-*methyl transferase; FkbH = FkbH-like domain; PCP = peptidyl-carrier protein domain; ST = sulfotransferase; C = condensation domain; A = adenylation domain; E = epimerization domain; MBOAT = membrane bound *O*-acyl transferase; PT = prenyltransferase; RO = Rieske oxygenase; GT = glycosyltransferase; 1,3-BPG = 1,3-bisphosphoglycerate; Agma = agmatine; Abn = azabicyclononane; Ile = isoleucine; Aaep = 1-amidino-3-(2-aminoethyl)-3-pyrroline (Aaep).

### Bioactivity Characterization

Prior investigations showed that structurally related compounds from the aeruginosin family have inhibitory effects on trypsin within the nanomolar range.^26,52^ In order to confirm the binding and categorize the bioactivity of our isolated suomilides to trypsin, we first performed a fluorescence-based activity assay, that revealed a highly potent concentration-dependent effect of suomilide B to trypsin (Figure 6A). At the highest concentration of 25 nM, the fluorescence signal remained almost constant at a low level, indicating complete inhibition of the reaction. In contrast, the lowest concentration of suomilide B at 0.1 nM resulted in fluorescence values slightly below the blank control, suggesting minimal residual activity. Furthermore, the IC_50_ values of the isolated compounds were determined to be 0.3 nM ± 0.05 for suomilide B, 8.4 nM ± 0.58 for suomilide D, 0.7 nM ± 0.05 for suomilide G (**1**) and 1.4 nM ± 0.14 for suomilide H (**2**) (Figure 6B; Table 2).

**Figure 6:**
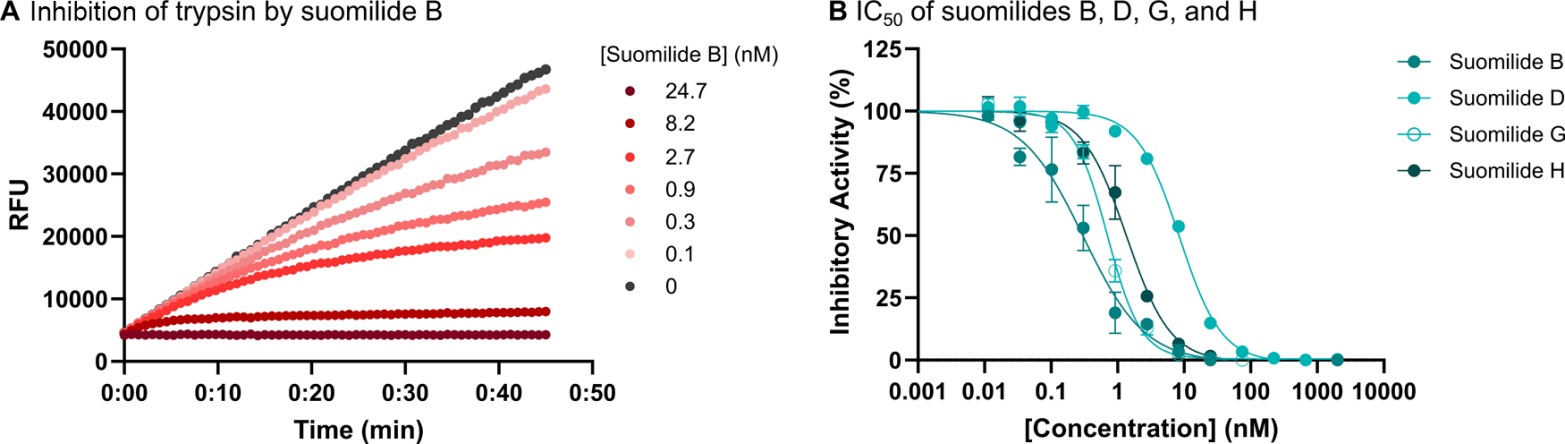
Inhibition of trypsin (100 pM in the assay) by suomilides. (A) Biphasic progress curves were observed for all compounds, as exemplified by the inhibition of trypsin by suomilide B (Table S4). (B) Determination of the IC_50_ values for the four suomilides. The measurements were performed in the presence of *N*-*p*-Tosyl-Gly-Pro-Arg-AMC (4.0 μM, *K*_M_ = 12 μM). The measured data (*n* = 3) were fitted to the 4-parameter logistic model. For better comparison between the different measurements, the velocity, determined as relative fluorescence units (RFU)/sec, was converted into percentage activity (Table S5).

**Table 2:** Determined IC_50_ Values for Suomilides B, D, G, and H

compound	IC_50_ value (nM)
suomilide B	0.3 ± 0.05
suomilide D	8.4 ± 0.58
suomilide G (**1**)	0.7 ± 0.05
suomilide H (**2**)	1.4 ± 0.14

Of all the tested compounds, suomilide B is the strongest trypsin inhibitor. Notably, this congener is produced in the largest quantity by *Nostoc* sp. KVJ20 (see Figure S3) indicating an optimized production of the most potent structure.

While we could successfully apply native metabolomics for the discovery of two new bioactive suomilides G (**1**) and H (**2**), one limitation of the study is the limited throughput of extracts that can be screened against buffer-exchanged trypsin, due to autolysis. This limitation may be mitigated by adding competitive trypsin inhibitors prior to native metabolomics experiments, which could also allow better preselection of selective and high-affinity binders targeting the orthosteric site. Future developments are expected to integrate binding detection with direct readouts of enzymatic activity, allowing activity-based screening and prioritization of functionally relevant inhibitors. Furthermore, native metabolomics is broadly applicable beyond enzymatic targets and can be extended to diverse protein classes, including nonenzymatic and heteromeric proteins, as demonstrated previously for CutA and related systems.^6,56^ In addition to protein–ligand interactions, the approach enables alternative discovery modes such as siderophore identification by substituting protein targets with defined metal ions to be chelated.^57,58^

## Conclusions

In this study, a native metabolomics approach tailored for trypsin-targeting cyanobacterial extracts enabled the identification of two previously unreported members of the suomilide family. We demonstrate the efficiency of the native metabolomics approach for screening trypsin, revealing a general trend for suomilides to exhibit strong trypsin inhibition. Suomilides B, D, G, and H inhibit trypsin at low nanomolar to high picomolar range, highlighting their high potency. Thus, our study presents a fast and rational screening approach to discover and link new natural products with potent and selective bioactivities.

## Experimental Section

### General Experimental Procedures

Optical rotations were measured with a Jasco P-2000 polarimeter. All NMR spectra were recorded in DMSO-*d*_6_ at 30 °C using a Bruker Ascend 600 spectrometer equipped with a Prodigy cryoprobe operating at 600 MHz (^1^H) and 150 MHz (^13^C). NMR spectra were processed using MestReNova 14.2 and referenced to residual solvent signals with resonances at δ_H/C_ 2.50/39.5. HPLC was carried out either on a Waters Alliance HPLC system equipped with a 2695 separation module, a 2998 PDA detector and an Acquity QDA mass detector or on a Waters HPLC system consisting of a 600E pump, a 996 PDA detector and a 717 plus autosampler.

### Cultivation of *Nostoc* sp. KVJ20

*Nostoc* sp. KVJ20 was grown in Fernbach flasks containing 500 mL BG11-_0_, which was prepared according to Culture collection of Algae and Protozoa (Allen & Stanier, 1968).^59^ The cultivation was performed at room temperature under constant illumination between 30 and 40 μmol/m^2^/s. By weekly splitting of cultures (250 mL culture +250 mL fresh medium), a total of 80 flasks was obtained yielding a final volume of 40 L. After 1 week of cultivation the cells were harvested by centrifugation and freeze-dried for at least 24 h yielding 16.3 g of dried cells.

### Extraction and Isolation

The freeze-dried cells were extracted stepwise with 100% methanol, 50% methanol *aq.,* 100% ddH_2_O and again 100% methanol by applying 5 min of sonification in each extraction step. The respective supernatants were collected after centrifugation. Evaporation of the pooled supernatants yielded 5.5 g of raw extract. The raw extract was dissolved in 100% methanol for purification with reversed-phase flash chromatography (Grace Reveleris X2). The column used was a FlashPure EcoFlex C_18_ with 220 g sorbent mass and 50 μm spherical particles. The mobile phases consisted of water (solvent A) and methanol (solvent B). A stepwise gradient from 70–100% solvent B over 30 min at a flow rate of 50 mL/min was performed and hold for additional 160 min at 100% solvent B. The fractions from each run were analyzed by LC-MS and suomilide containing fractions were pooled and evaporated resulting in 665.9 mg of a suomilide-enriched subfraction, which was redissolved for preparative HPLC. For HPLC purification a Waters XBridge Shield C_18_ column (150 × 4.6 mm, 5 μm) was used and a gradient over 30 min at a flow rate of 1 mL/min from 10–100% solvent B was employed followed by 5 min at 100% solvent B. Finally, solvent B was reduced to 10% over 1 min followed by a 14 min hold at 10% solvent B for equilibration. Optimal detection conditions were found at 210 nm. The resulting fractions were analyzed for compound identity and purity by LC-MS. 4.1 mg suomilide D were obtained while the remaining fractions underwent an additional purification step. A second purification step was performed on the same Waters system with a Macherey-Nagel Nucleoshell C_18_ plus column (250 × 4.6 mm, 5 μm, spherical). A flow rate of 0.8 mL/min was established, with acetonitrile replacing the methanol previously used as solvent B. Apart from that, the method employed was analogous to that used for the first purification step. A total of 4.0 mg suomilide B, 0.5 mg suomilide H (**2**), 2.0 mg suomilide G (**1**) and additional 0.9 mg suomilide D were collected.

#### Suomilide G (**1**)

colorless film (2.0 mg); [α]_D_^25^ +44.2 (*c* 0.16; MeOH); HR-ESI mass measurement *m*/*z* 1007.4246 (calcd for C_41_H_67_N_8_O_19_S, [M + H]^+^ 1007.4238). NMR data see Tables 1 and S2.

#### Suomilide H (**2**)

colorless film (0.5 mg); [α]_D_^25^ +7.5 (*c* 0.03; MeOH); HR-ESI mass measurement *m*/*z* 1105.4990 (calcd for C_47_H_77_N_8_O_20_S, [M + H]^+^ 1105.4969). NMR data see Table 1.

### Amino Acid Analysis of FDLA Derivatives

0.6 mg of compound **1** was hydrolyzed at 110 °C for 16 h in 6 m HCl (0.5 mL). The hydrolysate was dried with a stream of nitrogen. The dried residue was solved in 100 μL NaHCO_3_ (0.5 m) and 200 μL of 1% specl-FDLA (*N* ^α^-(5-fluoro-2,4-dinitrophenyl)-l-leucinamide) in acetone, respectively. The reaction mixture was heated at 70 °C for 45 min. The reaction was quenched by the addition of 25 μL of 2 m HCl. The solution was diluted 20-fold with acetonitrile (ACN). Five μL of the L-FDLA derivative was analyzed by HPLC ESI/MS. A first HPLC was performed using a Waters XBridge C_18_ column (100 × 2.1 mm; 3.5 μm; flow: 0.3 mL; gradient: 90/10 H_2_O/ACN (both 2mm NH_4_Ac) to 80/20 H_2_O/ACN (both 2mm NH_4_Ac) within 10 min, and then to 40/60 H_2_O/ACN (both 2 mm NH_4_Ac) within 10 min). The retention times were: 18.8 min (d-*allo*-isoleucine; d-isoleucine; hydrolysate); 15.7 min (l-*allo*-isoleucine; l-isoleucine). Therefore, the isoleucine residue in 1 was identified as either to be d-*allo*-isoleucine or d-isoleucine. To discriminate between these two diastereomers, a second HPLC using a chiral Phenomex Lux Cellulose-2 column (250 × 4.6 mm; 5 μm; flow 0.7 mL: isocratic for 20 min: 10/90 H_2_O/ACN (both 2mm NH_4_Ac) was performed. In this experiment the retention times were: 10.6 min (d-*allo*-isoleucine; hydrolysate); 10.8 min (d-isoleucine). The isoleucine residue in **1** finally was identified to be d-*allo*-isoleucine.

### Microflow LC-MS/MS Data Acquisition – Metabolomics

Analyses were performed using an Agilent 1260 Infinity II HPLC system with a quaternary G7104C pump, a G7167A Multisampler and a G7116A Multicolumn Thermostat (Agilent, Waldbronn, Germany) coupled via an ESI source to a Q-Exactive Plus quadrupole orbitrap mass spectrometer (Thermo Fisher Scientific, Bremen, Germany). A C_18_ reversed-phase core–shell microflow column (Kinetex C_18_, 150 × 1 mm, 1.7 μm particle size, Phenomenex, Torrance, USA) with column temperature of 60 °C was used for separation. Solvent A (H_2_O + 0.05% formic acid (FA)) and solvent B (acetonitrile (ACN) + 0.045% FA) were used as the mobile phase. The primary flow rate was set to 600 μL/min. This primary flow was splitted 1:10 using an ASI 620-PO10-11 flow splitter between the pump-outlet and the autosampler, resulting in an effective flow of 60 μL/min. Additionally, a makeup flow of 100 mM NH4Ac was applied with a flow rate of 100 μL/min using an additional isocratic pump module G1310A (Agilent, Waldbronn, Germany). The gradient applied was: 10–40% B between 0 and 8 min, followed by 40–99% B between 8 and 10 min, followed by a 3 min washout phase at 99% B and a 7 min re-equilibration phase at 10% B. MS/MS spectra were recorded in positive mode using data-dependent acquisition (DDA). Following each survey-scan, the top5 most abundant ions were selected for fragmentation. Survey-scans were performed with a resolution of 140,000 (@ *m*/*z* 200) using a mass range of 200–2000 *m*/*z* and fragment ion scans were recorded with a resolution of 17.500 (@ *m*/*z* 200), a stepped normalized collision energy of 25, 35, and 45 and a dynamic precursor exclusion of 10 s. Ions with unassigned charged states as well as charge states of 4–8 and higher than 8 were excluded from MS/MS acquisition, as well as isotope peaks.

### Microflow LC-MS/MS Data Acquisition – Native Metabolomics

The chromatographic conditions were exactly the same as described above for the Metabolomics runs. For MS, a spray voltage of 3 kV was applied with a S-lens RF level of 30 and a heated capillary temperature of 253 °C. The sheath gas flow rate was set to 40 L/min, the aux gas flow rate to 10 and the sweep gas flow rate to 0. The native metabolomics run was performed using all-ion fragmentation (AIF) mode with 15 V higher-energy collisional dissociation (HCD) with a resolution of 17,500 (@ *m*/*z* 200) and 5 microscans. Porcine trypsin (Sigma-Aldrich Chemie GmbH, Taufkirchen, Germany) was diluted to 40 μM in 100 mM NH_4_Ac buffer (pH 7.8) and infused via a syringe pump at a flow rate of 15 μL/min during native metabolomics measurements.

### Native MS Measurements

All native MS analyses were carried out using identical HPLC settings as for (Native)-Metabolomics runs, except for using an isocratic flow of 10% B instead of a gradient. The ESI-settings also were identical, except of recording 10 microscans instead of 5. Trypsin was analyzed at a concentration of 40 μM, with an infusion rate of 5 μL/min using a syringe pump. As a positive control, the inhibitor BAPA^44^ was used at equimolar concentrations, with the sample solution infused at 10 μL/min using a syringe pump.

### Native Metabolomics/Native MS Data Analysis

Data was analyzed with Xcalibur 2.2 and Unidec software.^60,61^ Unidec was used for deconvolution of the intact protein mass spectrometric data.

### LC-HR-MS/MS Data Acquisition

LC-MS/MS analysis for Feature-Based Molecular Networking was performed using a 1260 Infinity II HPLC system (Agilent, Germany) coupled to a Q-Exactive Plus Orbitrap mass spectrometer (Thermo Fisher Scientific, Germany) via an ESI source and equipped with a C_18_ reversed-phase column (EC Nucleoshell C_18_, 100 × 2 mm, 2.7 μm, Macherey-Nagel, Germany) at 25 °C column temperature. Water with 0.05% formic acid (FA) and acetonitrile with 0.045% FA served as mobile phases, with a flow rate of 200 μL/min. The gradient increased from 10–40% B over 8 min, then to 99% B over 2 min, followed by a 3 min wash and 5 min re-equilibration at 10% B. MS/MS spectra were acquired in positive mode using data-dependent acquisition (DDA). MS scans covered *m*/*z* 200–1200 at a resolution of 140,000 resolution (@ 200 *m*/*z*) with an AGC of 3e6 and 100 ms maximum injection time. MS/MS scans used 17,500 resolution (@ *m*/*z* 200), an AGC of 1e5, 150 ms maximum injection, an isolation window of 0.8 *m*/*z*, and stepped collision energies of 20, 30, and 40. Dynamic precursor exclusion was set to 10 s, and ions with unassigned or high charge states (4–8, >8) and isotopic peaks were excluded.

### File Conversion

LC-MS/MS data sets acquired in Thermo Scientific.RAW format were processed to mzML files with MSConvert (ProteoWizard).

### Feature-Based Molecular Networking

The converted MS data were preprocessed using MZmine 4.6.1 and the integrated MZWizard tool, which enabled automated extraction and alignment of features within a streamlined workflow.^45^ For mass detection, we applied noise thresholds of 5.00 for MS and 2.50 for MS2 to remove low-intensity background signals. Chromatograms were then constructed using a minimum of four consecutive scans, a minimum peak height of 5.0E4, and an *m*/*z* tolerance of 10 ppm.^62^ To improve signal quality and peak definition, chromatographic peaks were smoothed with a Savitzky-Golay filter using a window size of five data points.^63^ The local minimum feature resolver, ^13^C isotope, and isotopic peaks finder were added. Peak alignment across samples was carried out with the Join Aligner module, applying a RT tolerance of 0.1 min, and an *m*/*z* tolerance of 5 ppm. The preprocessed data were imported to GNPS2 and molecular networks were generated. The precursor and fragment ion mass tolerances were set to 0.02 Da. Edges were retained if they had a cosine score greater than 0.7 with at least six matched peaks and appeared within each node’s top 10 most similar neighbors. Molecular family size was limited to 100 by iteratively removing the lowest-scoring edges.^28,64^

### Molecular Network Visualization

GNPS2 outputs were exported to Cytoscape software (version 3.10.4) for analyzing and visualizing molecular networks.^65^

### MassQL Data Analysis

To identify the native metabolomics-guided trypsin inhibitors, we designed a MassQL query based on the characteristic MS2 fragments that all the binders in the high-resolution metabolomics run had in common. The characteristic MS2 fragment peaks are *m*/*z* 188.128, *m*/*z* 155.129, and *m*/*z* 109.029. The *m*/*z* tolerance was set on 0.005 Da and the minimum intensity percent was set on 5% of maximum peak in the spectrum. MassQL query: QUERY scaninfo­(MS2DATA) WHEREMS2PROD = 188.128 AND MS2PROD = 155.129 AND MS2PROD = 109.029:TOLERANCEMZ = 0.005:INTENSITYPERCENT = 5.

### Kinetic Measurements with Trypsin

Measurements with bovine trypsin (molecular weight 23.8 kDa, Thermo Fisher Scientific, Waltham, Massachusetts, USA) were performed using a Synergy H4 microplate reader (BioTek, now Agilent, Santa Clara, California, USA) in 50 mM TRIS-HCl buffer pH 8.0 containing 154 mM NaCl (λ_ex_ = 380 nm and λ_em_ = 460 nm) with 12 different inhibitor concentrations and a control in absence of inhibitor. Each well contained a total volume of 100 μL consisting of 70 μL of inhibitor dissolved in buffer, 20 μL of the substrate *N*-*p*-Tosyl-Gly-Pro-Arg-AMC (4 μM in the assay, *K*_M_ = 13.2 μM, Bachem AG, Bubendorf, Switzerland) dissolved in water and 10 μL of trypsin solution (100 pM in the assay). The enzymatic reactions were monitored for a total duration of 45 min. Owing to the biphasic, curved nature of the progress curves, only the final 15 min of the measurement were considered for kinetic evaluation. Reaction rates were determined by applying linear regression to the data points within this time interval. All measurements were conducted as triplicates in white 96-well plates (Greiner Bio-One GmbH, Frickenhausen, Germany) at room temperature.

## Supplementary Material



## Data Availability

NMR data of the newly described suomilides G and H have been uploaded to the Natural Products Magnetic Resonance Database (NP-MRD) under accession number NP0352385 for suomilides G (**1**) and NP0352386 for suomilides H (**2**). MS data was made publicly available through the MassIVE repository (MSV000100797). MS/MS spectra of suomilides G (**1**) (CCMSLIB00017456076) and H (**2**) (CCMSLIB00017456077) have been added to the GNPS library (gnps.ucsd.edu). Please find the FBMN job on GNPS Figure 3 here.

## Notes

Supplementary figures, tables, and NMR spectra of characterized compounds (PDF)
