# Supplementary material for: Native Metabolomics Unveils Suomilide Analogs with Potent Trypsin Inhibitory Activity
Source: J Nat Prod. 2026 Apr 1;89(4):1148–60. doi: 10.1021/acs.jnatprod.5c01380 (PMC13122637; doi:10.1021/acs.jnatprod.5c01380)
Supplement: Supplementary file 1 [file np5c01380_si_001.pdf]

Supporting Information

Native Metabolomics Unveils Suomilide Analogs with Potent Trypsin Inhibitory Activity

Amira Naimi#1, Christoph Ulbricht#2, Tung Lam Trinh[2], Stefan Kehraus[2], Teresa Marina Dreckmann[2], Uwe Linne[3], Kornelia Hardes[4], Raphael Reher*1, and Martin Baunach*2

[1]Institute of Pharmaceutical Biology and Biotechnology, Marburg University, Robert-Koch-Str. 4, 35037 Marburg, Germany

[2]Institute of Pharmaceutical Biology, University of Bonn, Nussallee 6, 53115 Bonn, Germany

[3]Department of Chemistry, Philipps-Universität Marburg, Hans-Meerwein-Straße 4, 35043 Marburg, Germany

[4]Fraunhofer Institute for Molecular Biology and Applied Ecology - Branch for Bioresources, Ohlebergsweg 12, 35392 Gießen, Germany

#These authors contributed equally.

*Co-corresponding authors.

Corresponding Authors

Raphael Reher - *Institute of Pharmaceutical Biology and Biotechnology, Department of Pharmacy, Philipps-University Marburg, Robert-Koch-Straße 4, 35037 Marburg, Germany*; Orcid ID: https://orcid.org/0000-0002-5858-1173; Email: raphael.reher@pharmazie.uni-marburg.de

Martin Baunach - *Institute of Pharmaceutical Biology, University of Bonn, Nussallee 6, 53115 Bonn, Germany*; Orcid ID: https://orcid.org/0000-0003-0822-1468; Email: mbaunach@uni-bonn.de

List of Figures

Figure S1. Porcine Trypsin. 3
Figure S2. SIRIUS/CANOPUS and manual MS2 analysis of putative suomilide D. 4
Figure S3. Network cluster of suomilides showing $-SO_3$ with intensity-scale nodes. 5
Figure S4. ^{1}H NMR spectrum of suomilide G (**1**) in DMSO-d_6 (600 MHz)..............................6
Figure S5. SMART 2.1 and DeepSAT analysis of compound **1**..6
Figure S6. ^{13}C NMR spectrum of suomilide G (**1**) in DMSO-d_6 (150 MHz). 7
Figure S7. ^{1}H-^{1}H COSY spectrum of suomilide G (**1**) in DMSO-d_6 (600 MHz).......................7
Figure S8. ^{1}H-^{13}C HSQC spectrum of suomilide G (**1**) in DMSO-d_6 (600 MHz). 8
Figure S9. ^{1}H-^{13}C HMBC spectrum of suomilide G (**1**) in DMSO-d_6 (600 MHz). 8
Figure S10. ^{1}H-^{1}H ROESY spectrum of suomilide G (**1**) in DMSO-d_6 (600 MHz). 9
Figure S11. ^{1}H NMR spectrum of suomilide H (**2**) in DMSO-d_6 (600 MHz). 9
Figure S12. ^{13}C NMR spectrum of suomilide H (**2**) in DMSO-d_6 (150 MHz).........................10
Figure S13. ^{1}H-^{1}H COSY spectrum of suomilide H (**2**) in DMSO-d_6 (600 MHz)...................10
Figure S14. ^{1}H-^{13}C HSQC spectrum of suomilide H (**2**) in DMSO-d_6 (600 MHz). 11
Figure S15. ^{1}H-^{13}C HMBC spectrum of suomilide H(**2**) in DMSO-d_6 (600 MHz). 11
Figure S16. ^{1}H NMR spectrum of suomilide B (**3**) in DMSO-d_6 (600 MHz). 12
Figure S17. ^{13}C NMR spectrum of suomilide B (**3**) in DMSO-d_6 (150 MHz).........................12
Figure S18. ^{1}H NMR spectrum of suomilide D (**4**) in DMSO-d_6 (600 MHz). 13
Figure S19. ^{13}C NMR spectrum of suomilide D (**4**) in DMSO-d_6 (150 MHz).........................13
Figure S20. Key COSY, HMBC and ROESY correlations of compound **1**. 14
Figure S21. Examples of NRPs incorporating conformational isomers of Ile.........................15

List of Tables

Table S1. Correlation of native metabolomics and conventional metabolomics runs for trypsin binders..16
Table S2. NMR Spectroscopic Data for Compound **1** (600 MHz, DMSO-d_6). 17
Table S3. Overview of NRPs from Figure S21 incorporating conformational isomers of Ile and their corresponding NRPS A domains. 18
Table S4. Data points used for biphasic progress curve for suomilide B. 19
Table S5. Data points used for IC_{50} curve for suomilide B, D, G, and H. 19

A Amino acide sequences

With propeptide: 231 amino acids - 24.409 kDa

FPTDDDDKIVGGYTCAANSIPYQVSLNSGSHFCGGSLINSQWVVSAAHCYKSRIQVRLGEHNIDVLE
GNEQFINAAKIITHPNFNGNTLDNDIMLIKLSSPATLNSRVATVSLPRSCAAAGTECLISGWGNTKSSG
SSYPSLLQCLKAPVLSDSSCKSSYPGQITGNMICVGFLEGGKDSCQGDSGGPVVCNGQLQGIVSW
GYGCAQKNKPGVYTKVCNYVNWIQQTIAAN

Activated trypsin: 223 amino acids - 23.475 kDa

IVGGYTCAANSIPYQVSLNSGSHFCGGSLINSQWVVSAAHCYKSRIQVRLGEHNIDVLEGNEQFINA
AKIITHPNFNGNTLDNDIMLIKLSSPATLNSRVATVSLPRSCAAAGTECLISGWGNTKSSGSSYPSLLQ
CLKAPVLSDSSCKSSYPGQITGNMICVGFLEGGKDSCQGDSGGPVVCNGQLQGIVSWGYGCAQK
NKPGVYTKVCNYVNWIQQTIAAN

B Mass of trypsin with intact disulfide bridges (oxidized cysteines)

Disulfide bonds:

15 ↔ 145, 33 ↔ 49, 117 ↔ 218,
124 ↔ 191, 156 ↔ 170, 181 ↔ 20

23463.538 Da (average)
23448.424 Da (monoisotopic)
23448.350 Da (deconvoluted, native MS)

C Deconvoluted mass of trypsin

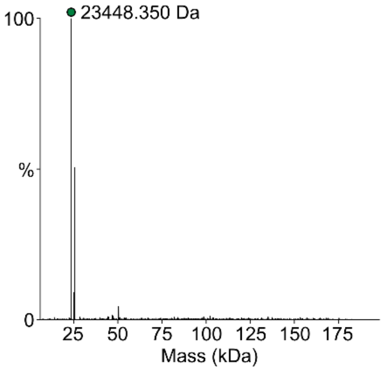

Figure S1. Porcine trypsin. **A** Amino acid sequence from porcine trypsin (*Sus scrofa*) with and without propeptide (octapeptide; marked in red) and masses in kDa. Uniprot: P00761. **B** Average and monoisotopic calculated masses of native porcine trypsin (activated trypsin with oxidized cysteines) vs native mass spectrometry deconvoluted mass in Da.[1] **C** Deconvoluted mass of trypsin.

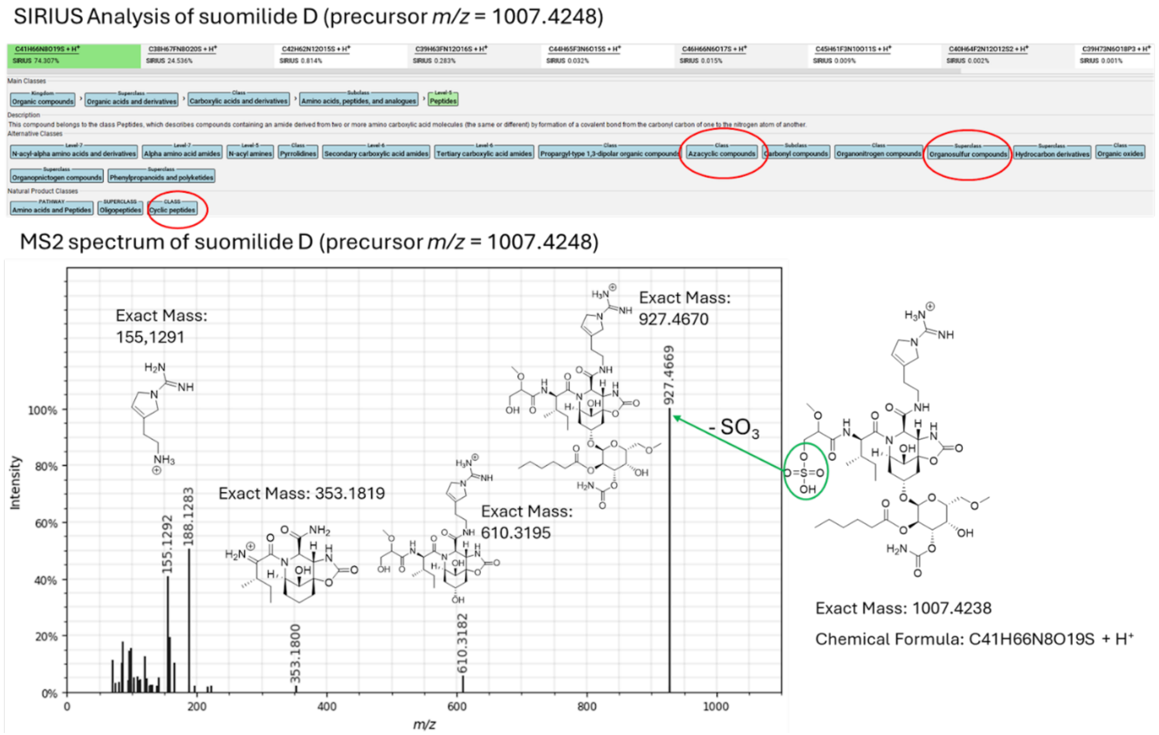

Figure S2. SIRIUS,[2] CANOPUS,[3] and manual MS2 analysis of putative suomilide D.

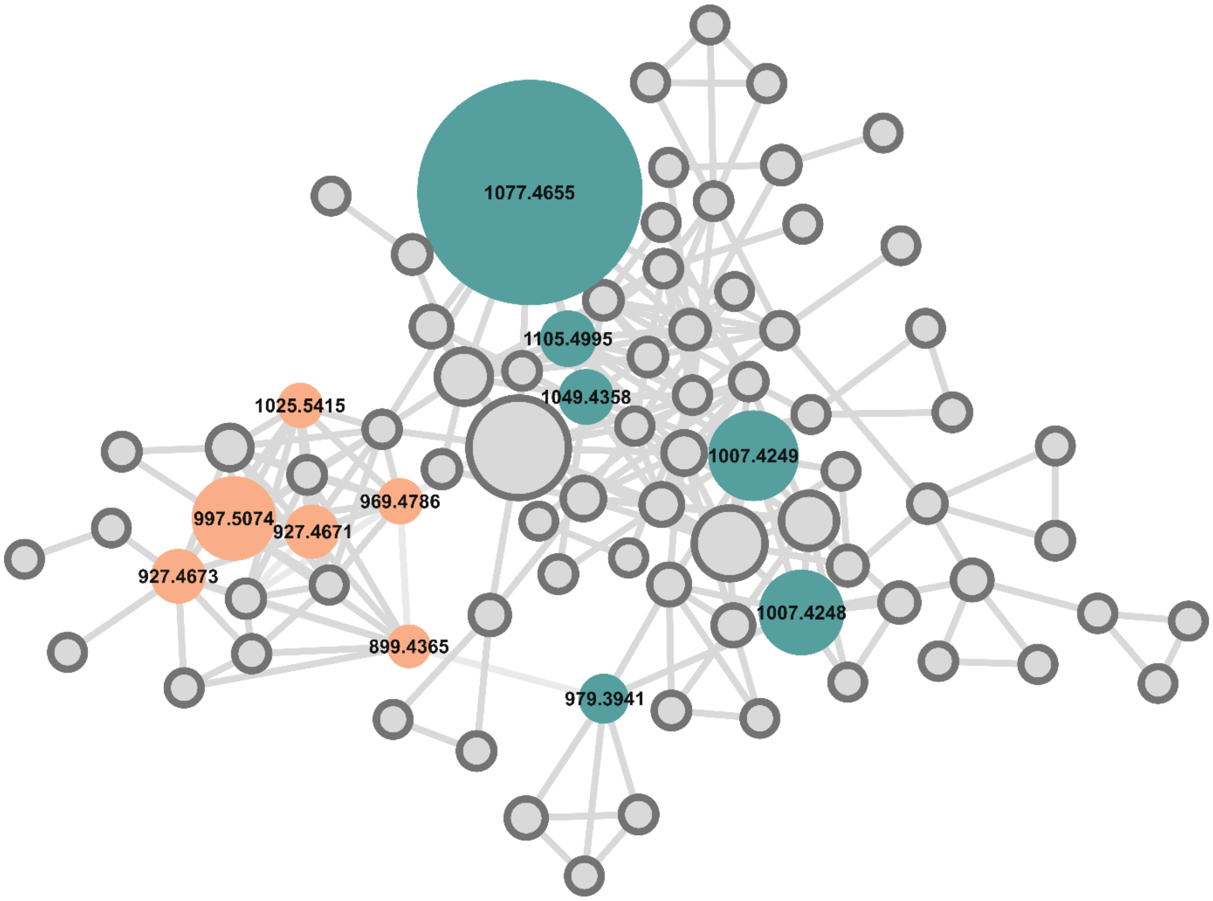

Figure S3. Network cluster of suomilides showing $-SO_3$ with intensity-scale nodes. Network cluster of suomilides with node sizes proportional to peak area. Annotated suomilides are shown in green, nodes representing sulfate losses in orange.

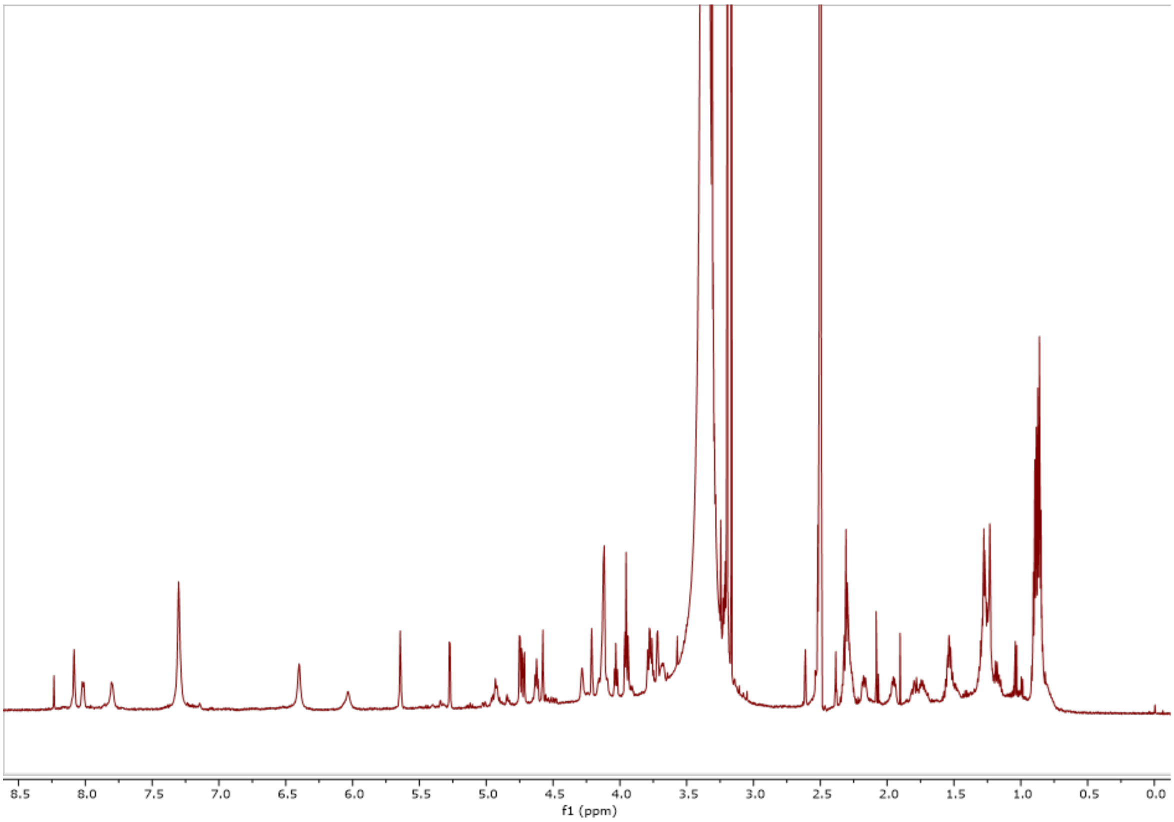

Figure S4. ^{1}H NMR spectrum of suomilide G (**1**) in DMSO-d_6 (600 MHz).

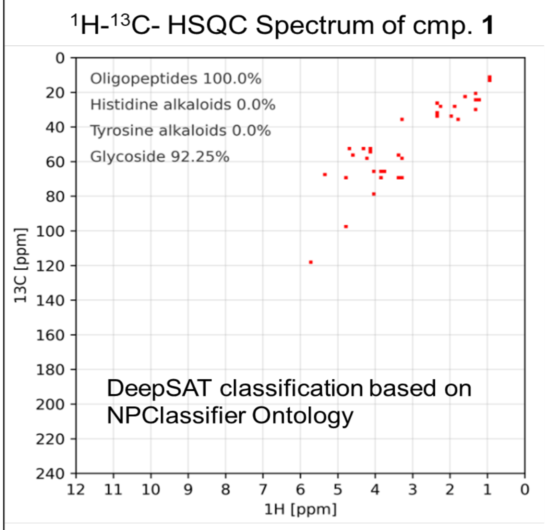

SMART 2.1: Top 3 Predictions

1. Banyaside A (cos. = 0.93)
2. Banyaside B (cos. = 0.88)
3. Suomilide (cos. = 0.83)

DeepSAT: Top 3 Predictions

1. Suomilide (cos. = 0.91)
2. Banyaside B (cos. = 0.90)
3. Banyaside A (cos. = 0.89)

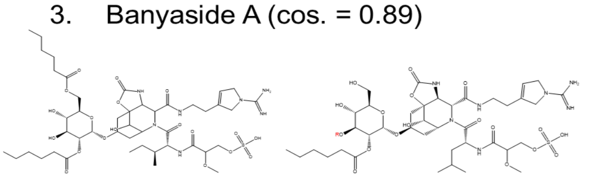

Suomilide

R = CONH2 = Banyaside A
R = H = Banyaside B

Figure S5. SMART 2.1[4] and DeepSAT[5] analysis of compound **1**.

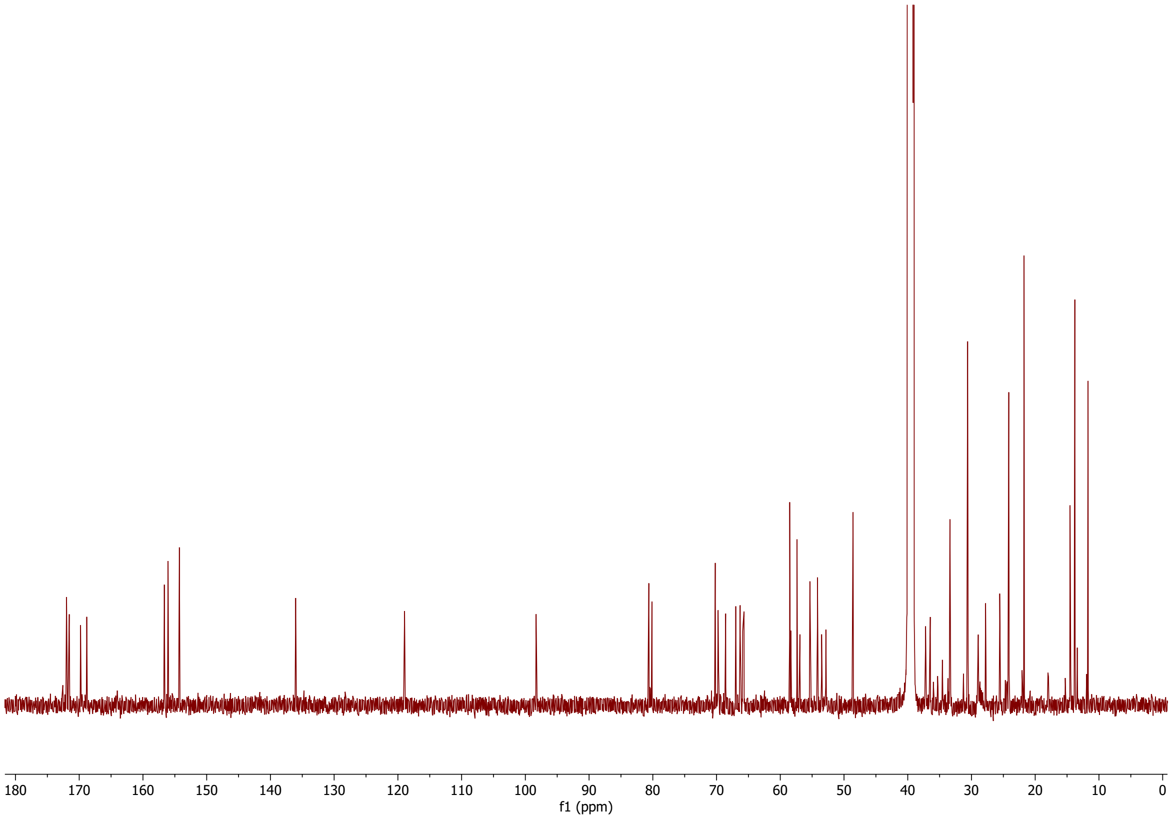

Figure S6. ^{13}C NMR spectrum of suomilide G (**1**) in DMSO-*d*$_6$ (150 MHz).

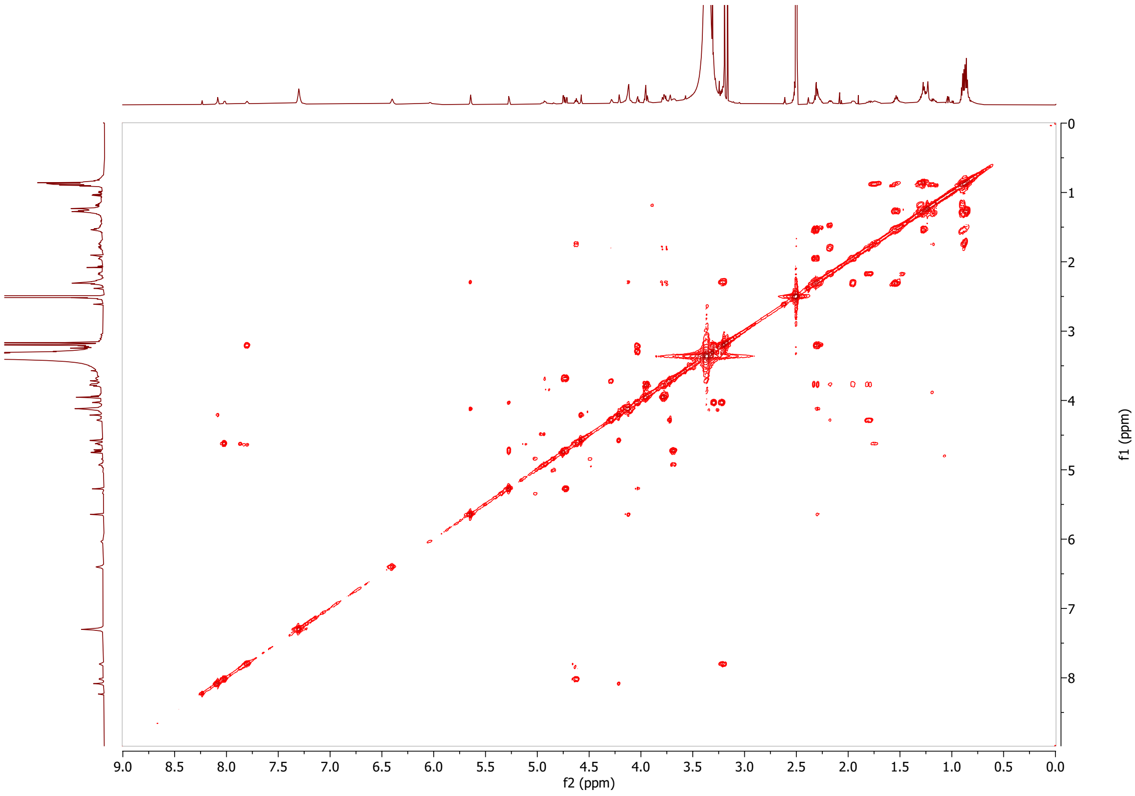

Figure S7: ^1H-^1H COSY spectrum of suomilide G (**1**) in DMSO-*d*$_6$ (600 MHz).

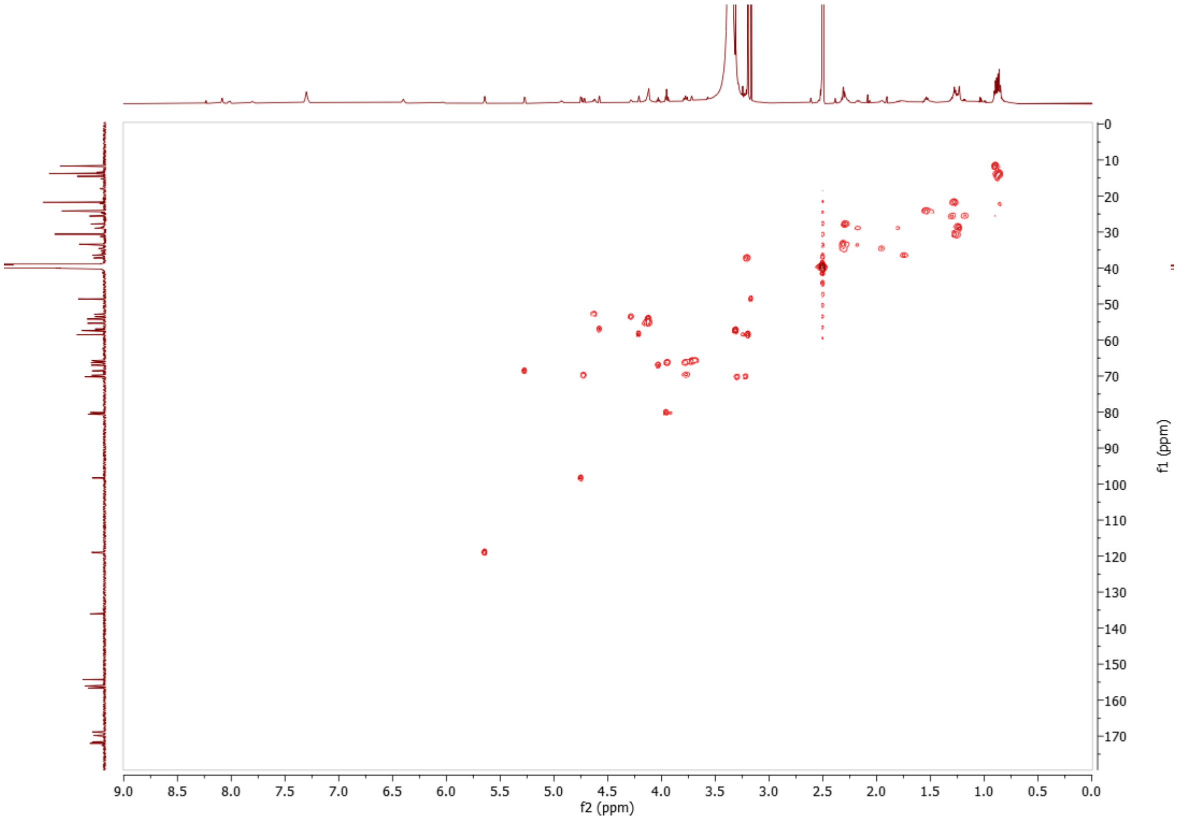

Figure S8: ^{1}H-^{13}C HSQC spectrum of suomilide G (**1**) in DMSO-d_6 (600 MHz).

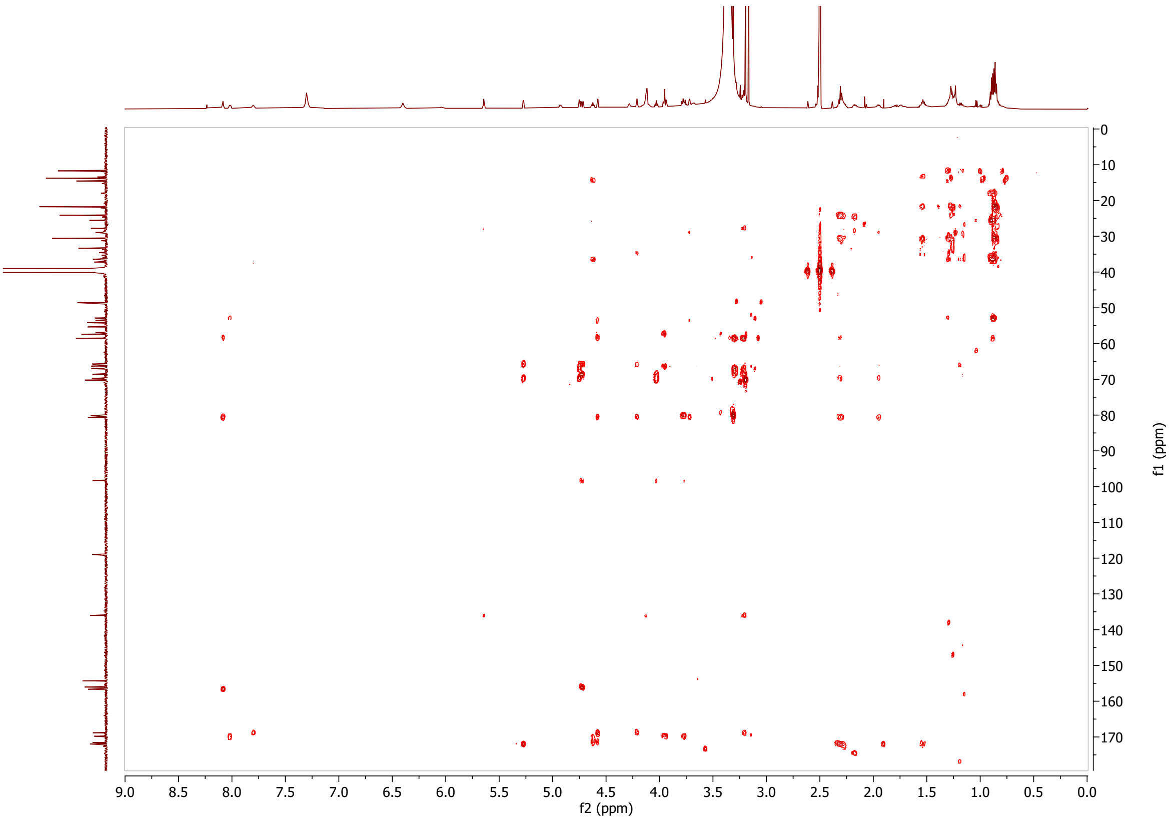

Figure S9: ^{1}H-^{13}C HMBC spectrum of suomilide G (**1**) in DMSO-d_6 (600 MHz).

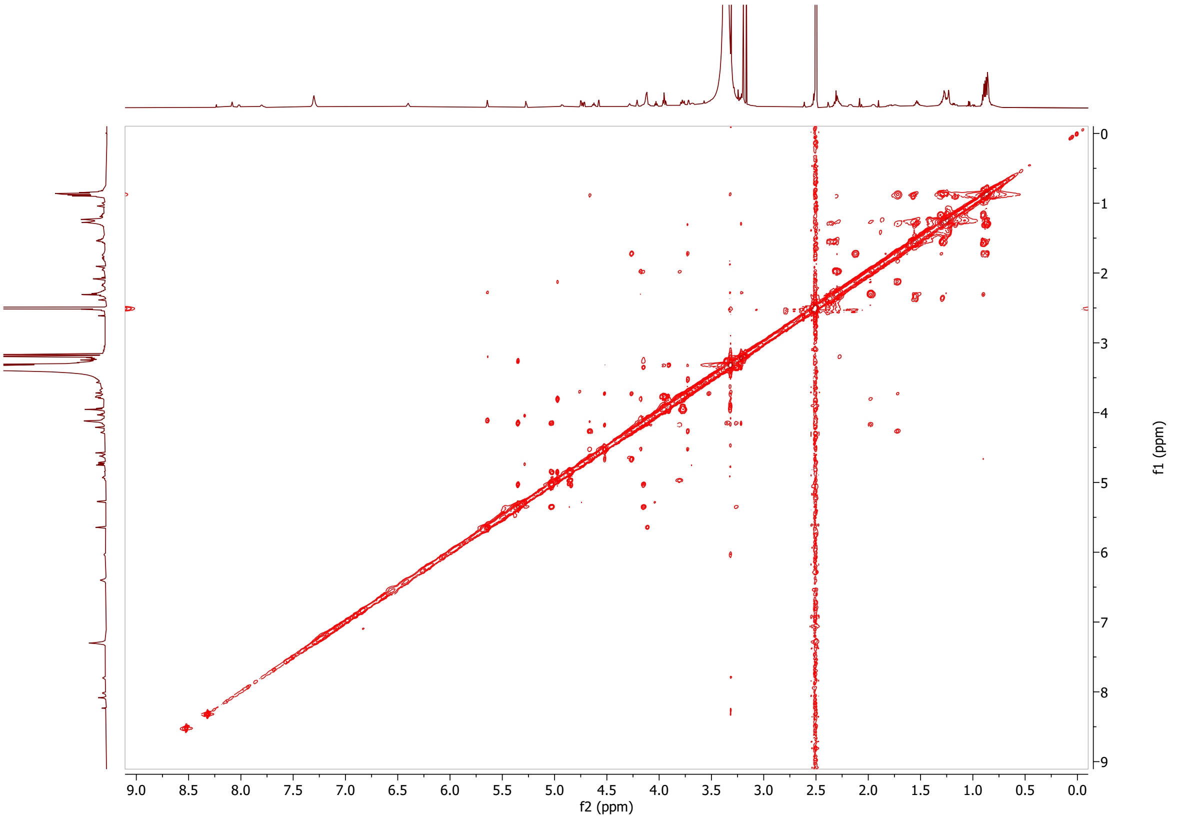

Figure S10: ^1H-^1H ROESY spectrum of suomilide G (**1**) in DMSO-*d*$_6$ (600 MHz).

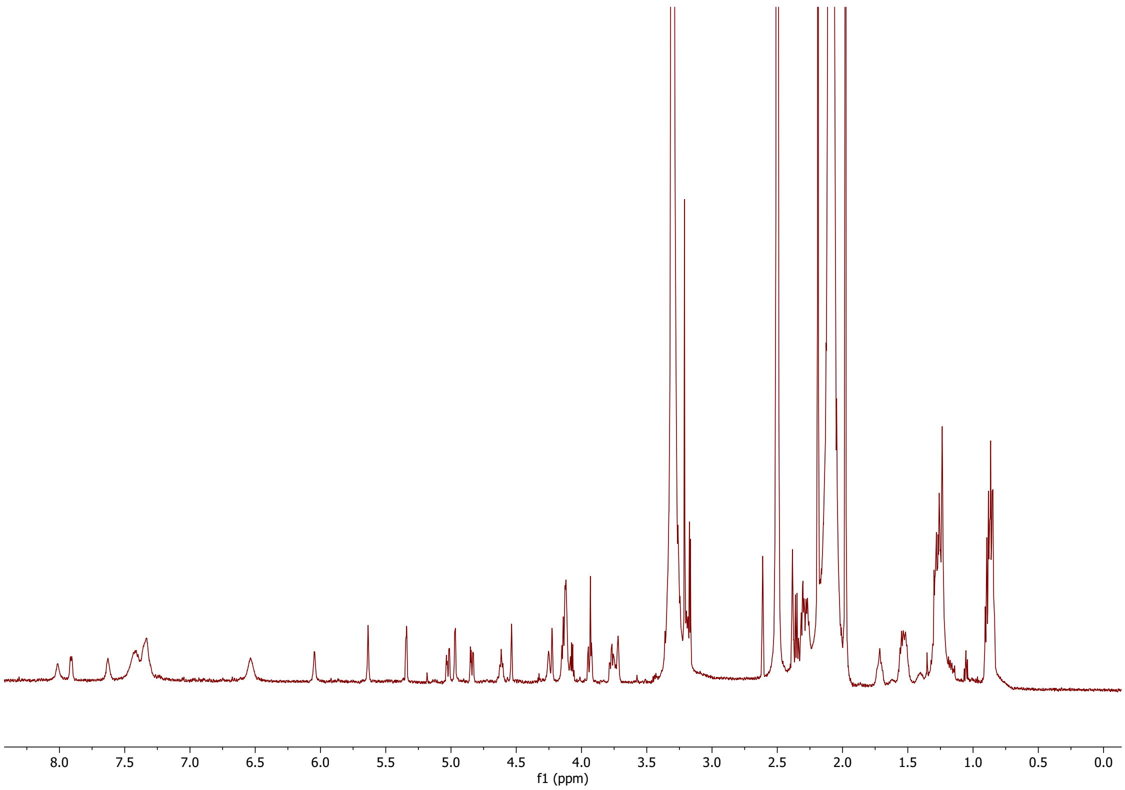

Figure S11. ^1H NMR spectrum of suomilide H (**2**) in DMSO-*d*$_6$ (600 MHz).

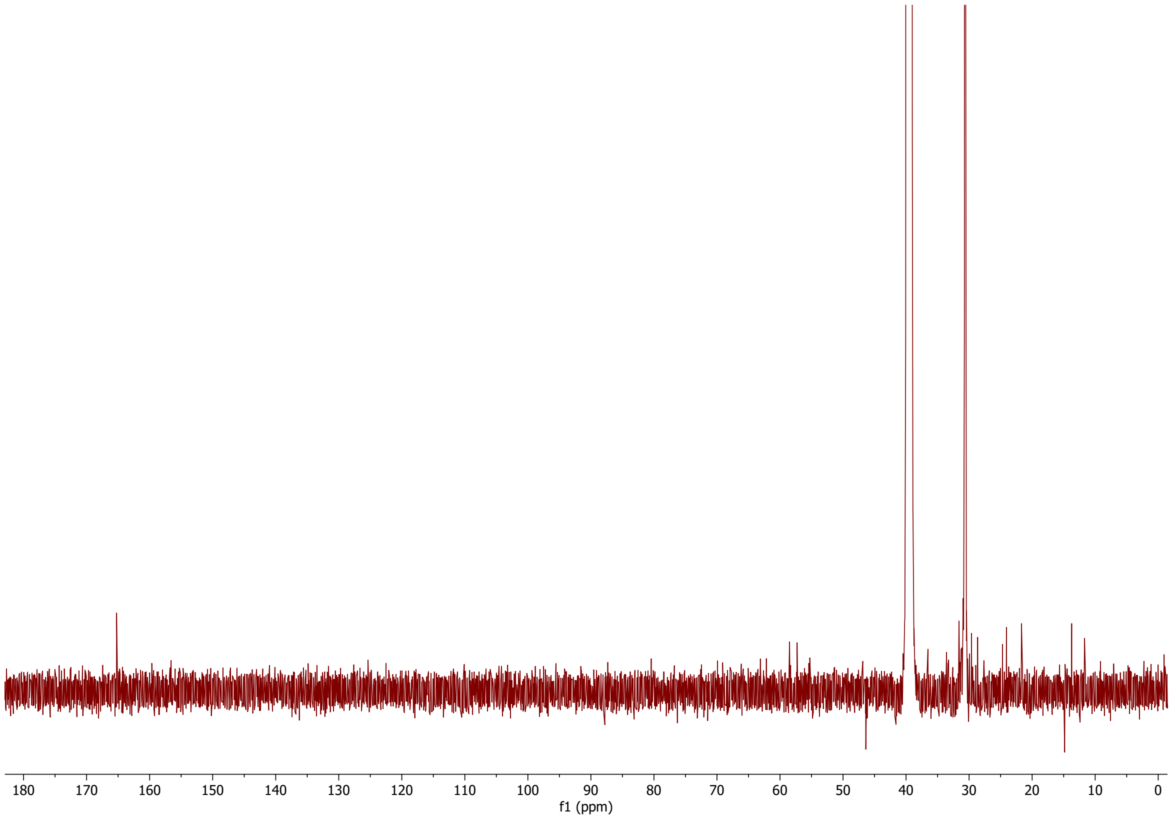

Figure S12. ^{13}C NMR spectrum of suomilide H (**2**) in DMSO-d_6 (150 MHz).

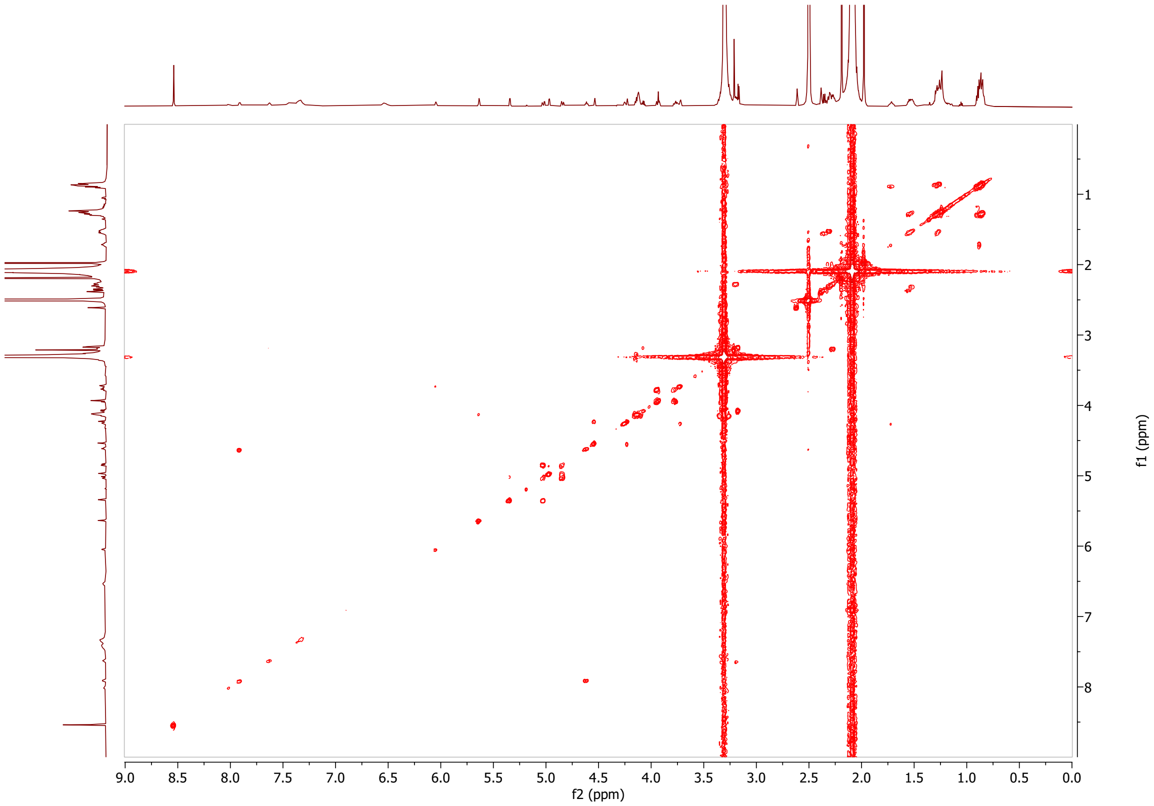

Figure S13: ^{1}H-^{1}H COSY spectrum of suomilide H (**2**) in DMSO-d_6 (600 MHz).

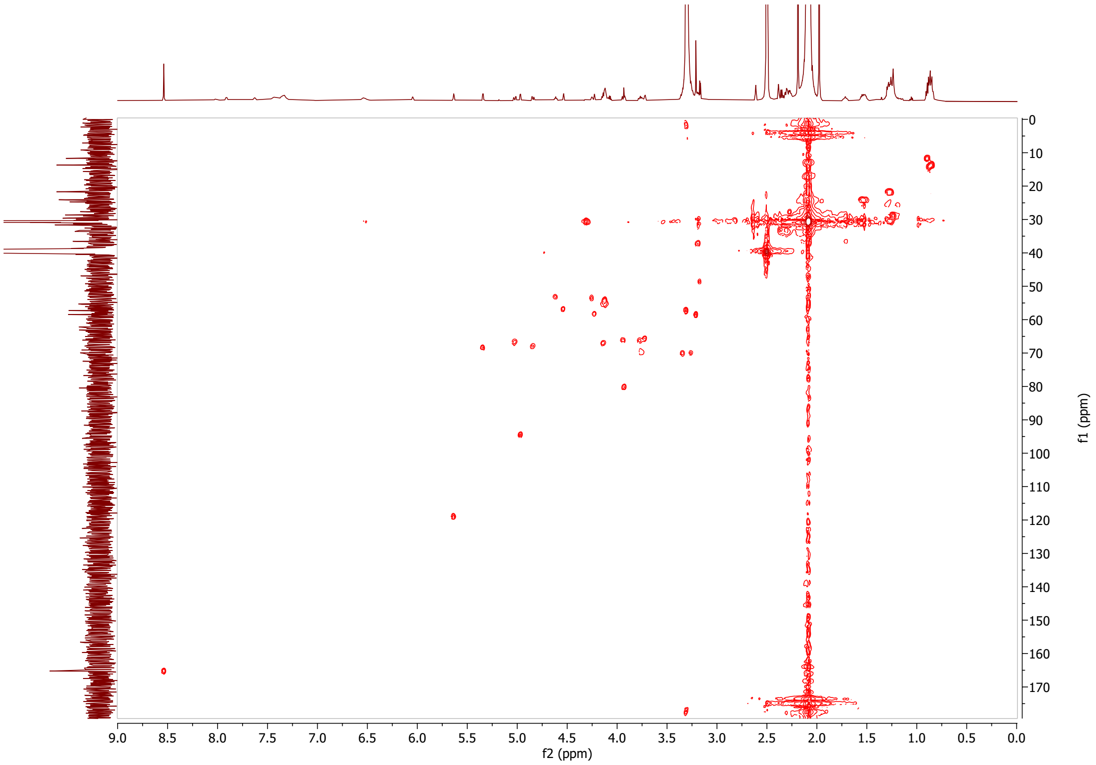

Figure S14. ^{1}H-^{13}C HSQC spectrum of suomilide H (**2**) in DMSO-d_6 (600 MHz).

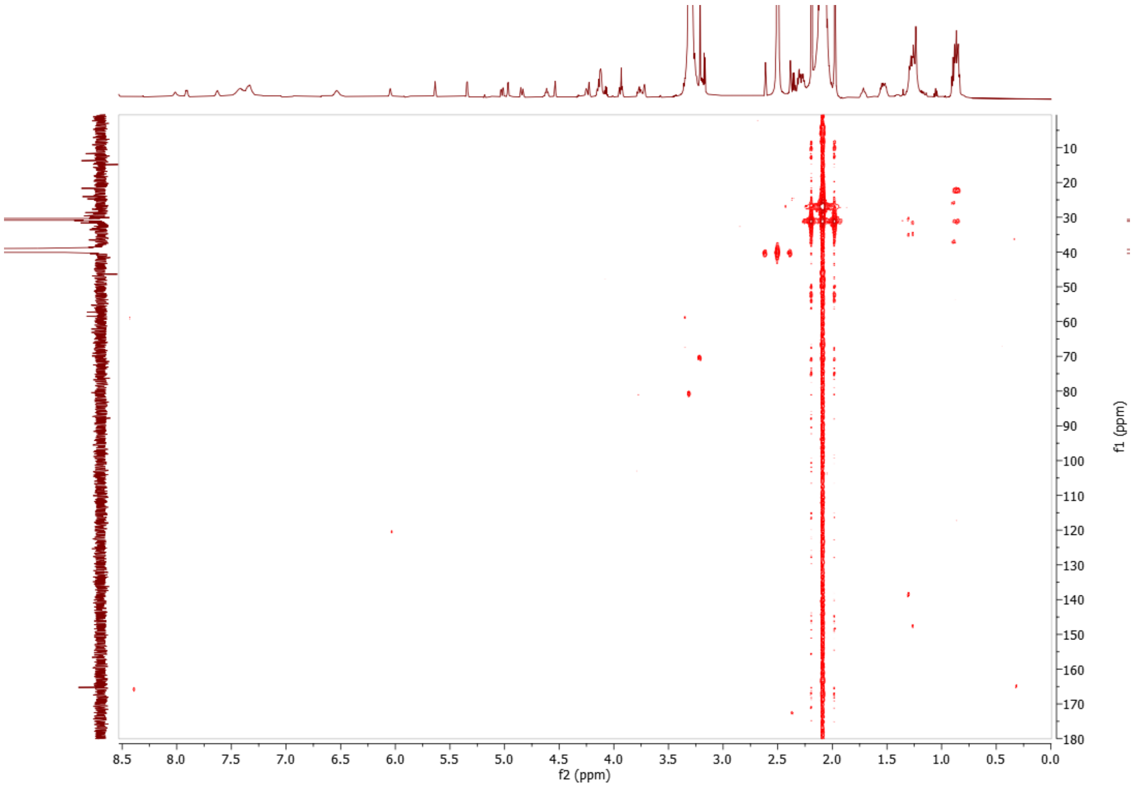

Figure S15: ^{1}H-^{13}C HMBC spectrum of suomilide H (**2**) in DMSO-d_6 (600 MHz).

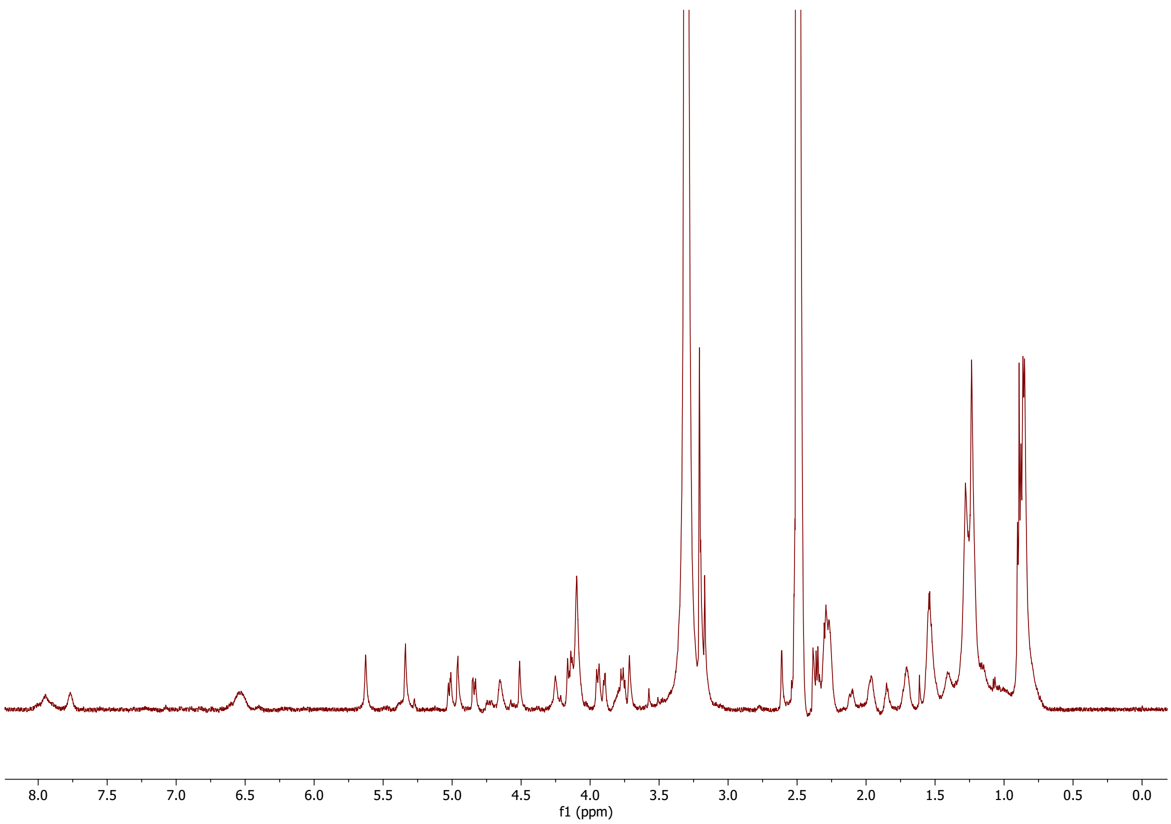

Figure S16. ^{1}H NMR spectrum of suomilide B (**3**) in DMSO-d_6 (600 MHz).

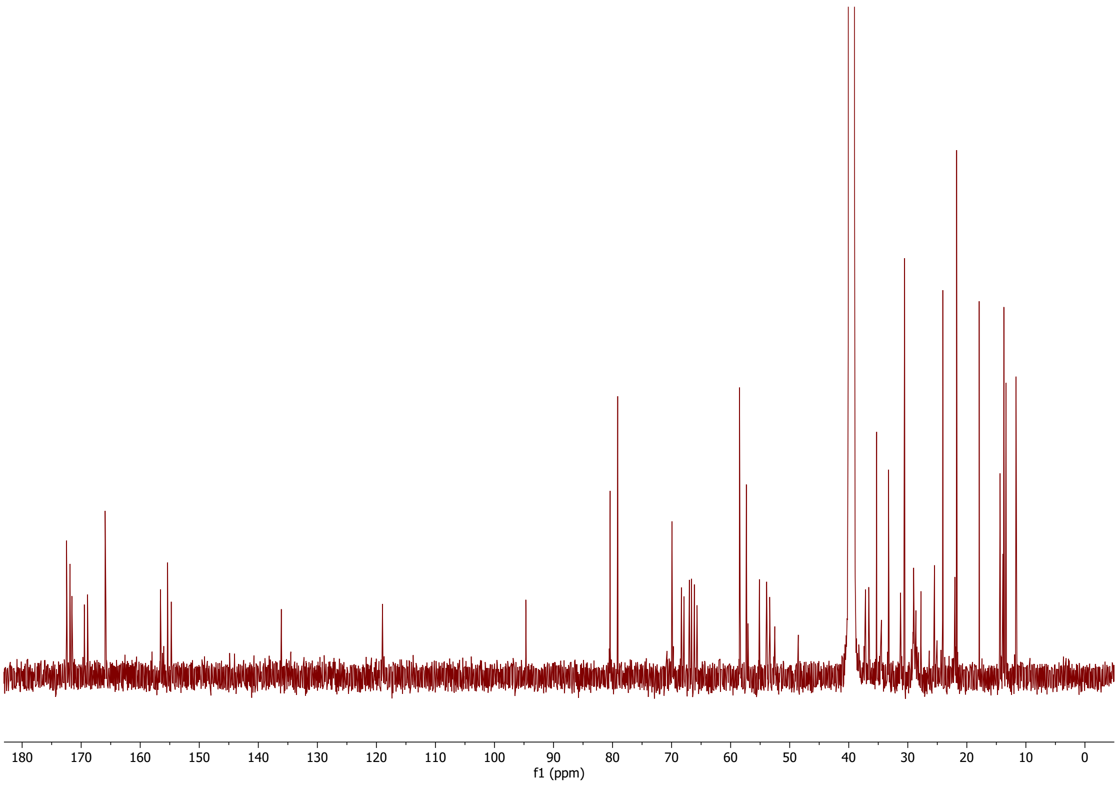

Figure S17. ^{13}C NMR spectrum of suomilide B (**3**) in DMSO-d_6 (150 MHz).

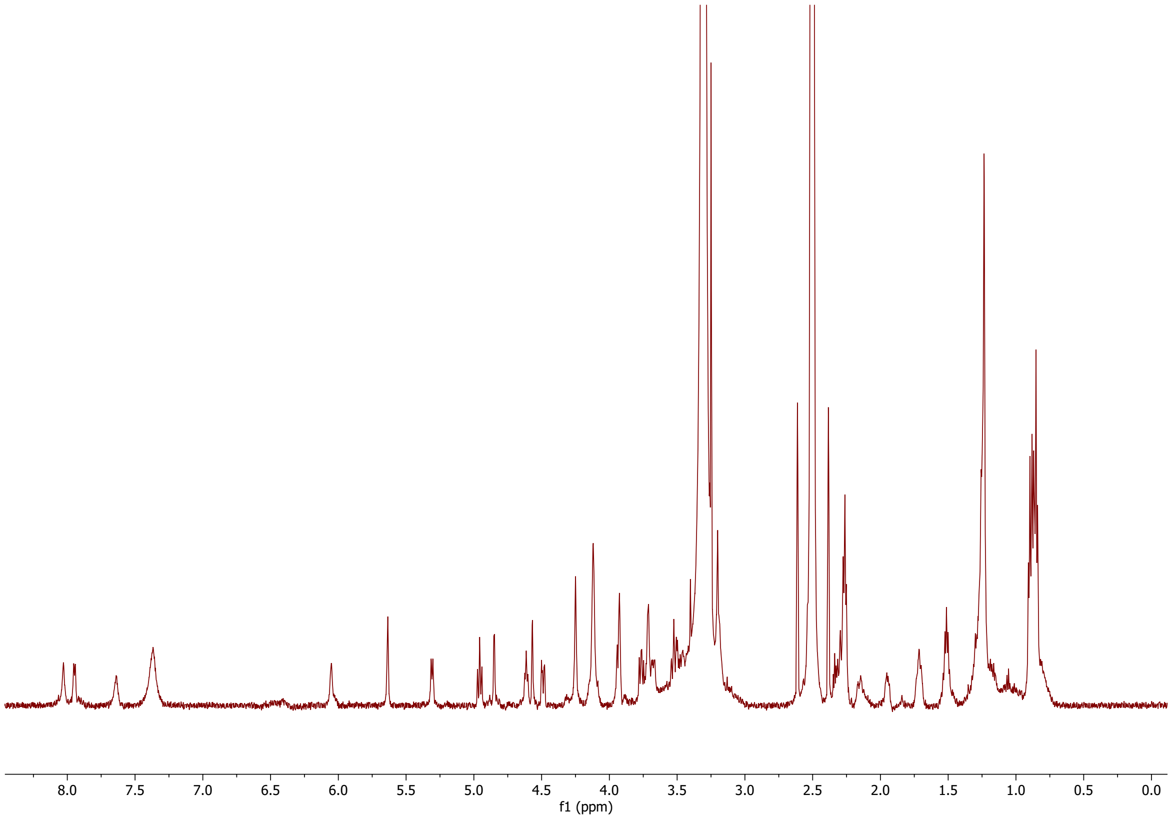

Figure S18. ^{1}H NMR spectrum of suomilide D (**4**) in DMSO-d_6 (600 MHz).

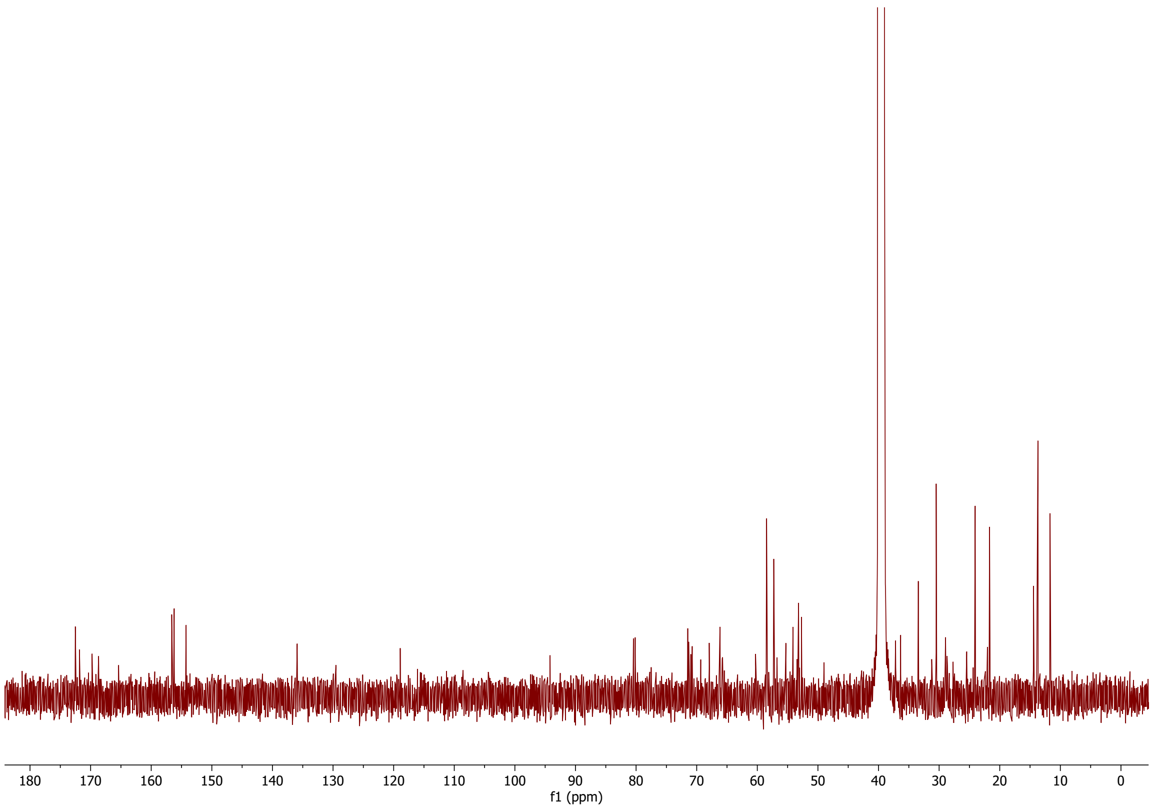

Figure S19. ^{13}C NMR spectrum of suomilide D (**4**) in DMSO-d_6 (150 MHz).

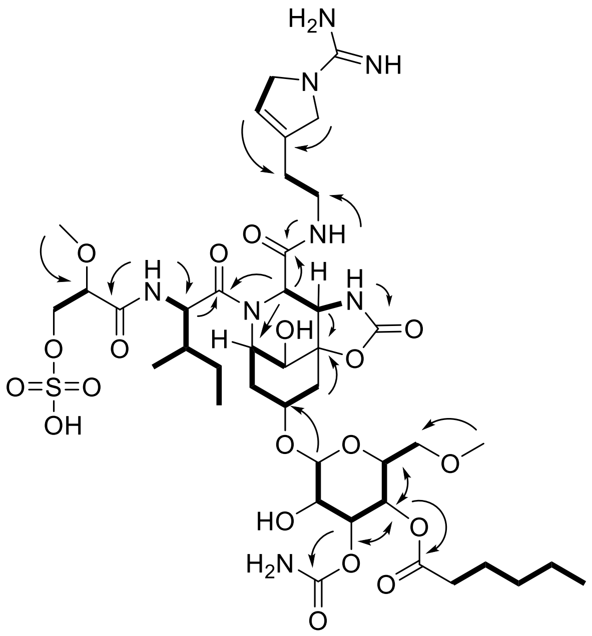

Figure S20. Key COSY (—), HMBC (⌒→) and ROESY (←⌒→) correlations of compound **1**.

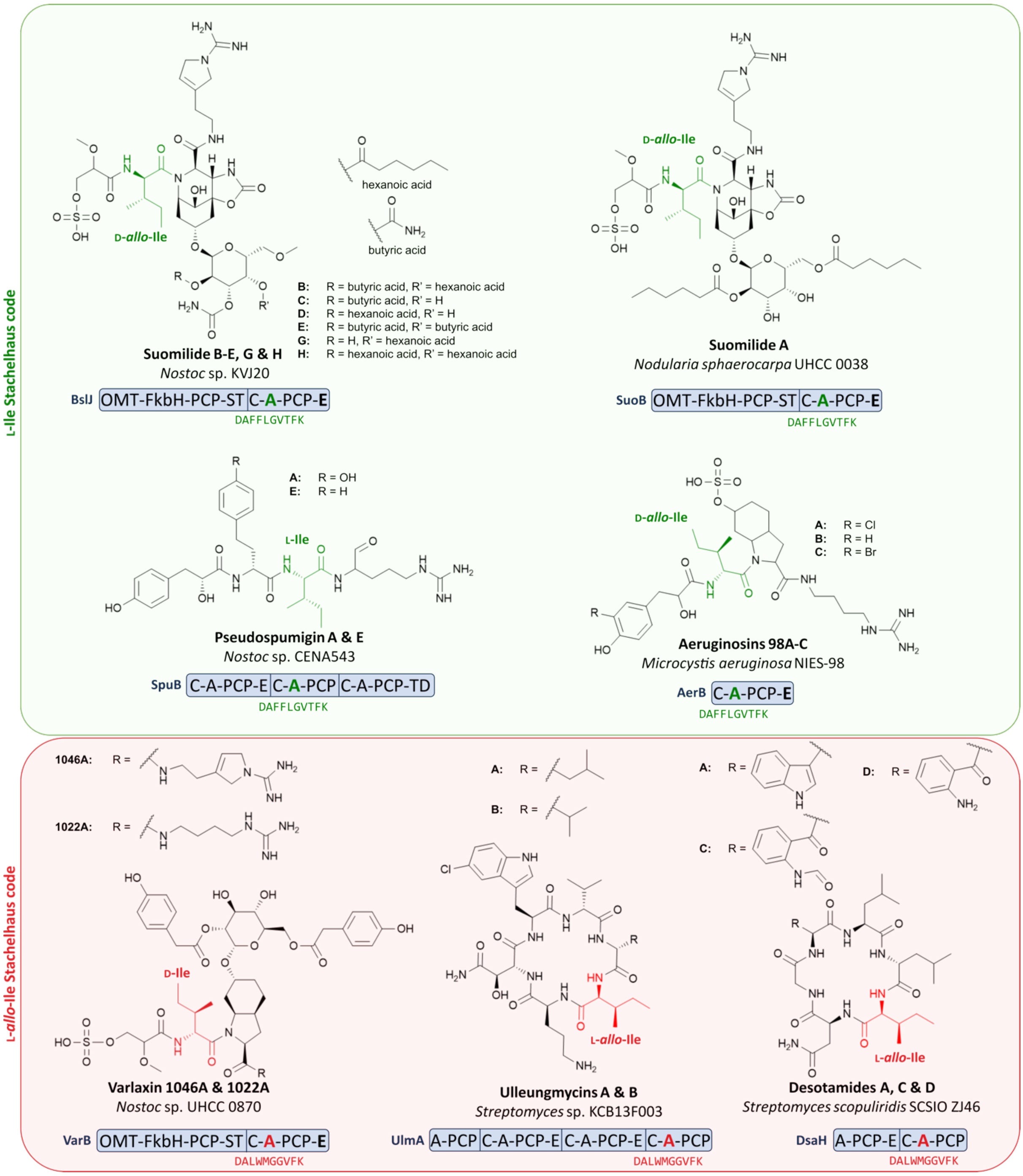

Figure S21. Examples of NRPs incorporating conformational isomers of Ile. The upper panel shows NRPs with A domains (green) recognizing L-Ile according to their Stachelhaus codes,[6] which, if followed by an epimerization (E) domain, are epimerized to D-*allo*-Ile. In contrast, NRPs in the lower panel contain A domains (red) specific for L-*allo*-Ile (sometimes also tolerant for L-Val), which, when followed by an E domain, yield D-Ile. Stachelhaus codes are shown below the respective A domains, and corresponding NRPS proteins are depicted in light blue. Accession numbers are listed in Table 1. OMT = *O*-methyl transferase; FkbH = FkbH-like domain; PCP = peptidyl-carrier protein domain; ST = sulfotransferase; C = condensation domain; A = adenylation domain; E = epimerization domain; TD = terminal reductase domain.

Table S1. Correlation of native metabolomics and conventional metabolomics runs for trypsin binders corresponding to Figure 2D-J.

Trypsin Binder	Retention time [min]	Native MS *Δm/z* (7+)	Native MS *Δm/z* (8+)	Native MS *Δm/z* (9+)	Averaged *Δm/z* (corrected for proton, +1.0073 Da)	Observed $[M+H]^+$ in metabolomics	Mass error [Da]
**Positive control BAPA**	n.d.	79.6349	69.5125	61.6991	557.2860	n.d.	n.d.
**1**	3.86–4.21	139.7072	122.4105	108.6859	979.4765	979.3921	0.0844
**2**	5.09–5.62	143.8460	125.7858	111.8761	1007.7051	1007.4223	0.2822
**3**	5.80–6.15	143.9475	125.9959	111.6957	1007.9610	1007.4234	0.5376
**4**	6.33–6.50	149.4893	131.0200	116.4249	1048.4770	1049.4344	0.9574
**5**	7.74–7.91	153.7373	134.5341	119.7803	1077.8262	1077.4643	0.3619
**6**	8.97–9.32	157.6205	138.1045	122.9906	1106.0389	1105.4956	0.5433

Explanatory Notes:

1. *Δm/z* values correspond to mass shifts observed for the protein–ligand complex at the indicated charge state in native MS runs.
2. Averaged *Δm/z* is corrected by adding a proton mass (+1.0073 Da) to allow direct comparison with the $[M+H]^+$ signal observed in the conventional metabolomics run.
3. Retention times indicate the elution range in the HPLC-native MS run.
4. Mass error is calculated as the difference between the averaged native MS mass and the observed $[M+H]^+$ from metabolomics.
5. n.d. = not detected.

Table S2. NMR Spectroscopic Data for Compound **1** (600 MHz, DMSO-d_6).

	Pos.		δ_H (J in Hz)	δ_C	COSY	HMBC	ROESY
Mgs	1	CH_2	a: 3.77, m	66.3	1b	2	1b
			b: 3.95, m		1a		1a
	2	CH	3.96, m	80.1		1, 3	
		OCH_3	3.31, s	57.4		2	
	3	C		169.8			
allo-Ile	4	NH	8.02, brd, 7.0		5	3, 5	
	5	CH	4.62, t, 7.0	52.8	4, 6	3, 6, 7, 10	18
	6	CH	1.74, m	36.5	5, 7		7
	7	CH_3	0.87, d, 7.5	14.5	6, 8a/b	5, 6, 8	6
	8	CH_2	a: 1.18, m	25.6	7, 9		
			b: 1.30, m		7, 9		
	9	CH_3	0.90, t, 7.4	11.7	8a/b	6, 8	
	10	C		171.6			
Abn	11	N					
	12	CH	4.58, d, 2.0	56.9	13	10, 13, 16, 18, 22	17
	13	CH	4.21, brs	58.4	12, 14	16, 17, 22	
	14	NH	8.08, s		13	13, 15, 16	
	15	C		156.7			
	16	C		80.6			
	17	CH	3.71, brs	65.7	OH-17, 18		12
		OH	6.03, brs		17		
	18	CH	4.28, brs	53.5	17, 19a		5, 17
	19	CH_2	a: 1.80, m	28.9	18,19b, 20		19b
			b: 2.17, m		18,19a, 20		19a
	20	CH	3.76, m	69.7	19a/b, 21a/b		
	21	CH_2	a: 1.95, m	34.6	20, 21b	16, 20	21b
			b: 2.30, m		21a	16	21a
Aaep	22	C		168.8			
	23	NH	7.80, brs		24	22, 24	24
	24	CH_2	3.20, m	37.2	23, 25		
	25	CH_2	2.29, m	27.8	24, 27, 30		
	26	C		136.0			
	27	CH	5.64, s	118.9	25, 28	25, 26	25, 28
	28	CH_2	4.12, m	54.2	27		27
	29	N					
	30	CH_2	4.12, m	55.4	25	26	
guanidine	31	C		154.3			
	32/32`	NH/NH_2	7.30, brs				
Gal	33	CH	4.75, d, 3.9	98.3	34	20, 37	
	34	CH	3.68, m	65.9	33, OH-34, 35		
		OH	4.92, brs		34		
	35	CH	4.72, dd, 3.5, 10.8	69.8	34, 36	34, 36, 46	36
	36	CH	5.27, dd, 1.1, 3.5	68.6	35, 37	34, 35, 40	35, 37, 38
	37	CH	4.02, brt, 6.5	67.0	36, 38a/b	36, 38	36, 38
	38	CH_2	a: 3.22, m	70.2	37, 38b	39	37
			b: 3.29, m		37, 38a	39	37
	39	OCH_3	3.19, s	58.5		38	
HA-1	40	C		172.0			
	41	CH_2	2.30, m	33.4	42	40, 42, 43	42
	42	CH_2	1.54, m	24.2	41, 43	40, 43, 44	41
	43	CH_2	1.30, m	30.6	42	44	
	44	CH_2	1.27, m	21.7	45	43	45
	45	CH_3	0.85, t	13.8	44	43, 44	44
	46	C		156.1			
	47	NH_2	6.40, brs				

Table S3. Overview of NRPs from Figure S21 incorporating conformational isomers of Ile and their corresponding NRPS A domains. The table provides information on the biosynthetic pathways (with MIBiG[7] accession numbers or literature references) and the proteins containing the respective Ile-recruiting A domains. For each A domain, the position within the protein, the PARAS-predicted Stachelhaus code,[6,8] and the resulting incorporated amino acid (AA) in the peptide are shown.

Pathway			Protein		A Domain		
**Compounds**	**Producer Organism**	**MIBiG Accession Nr. or reference**	**Name**	**NCBI Accession Nr.**	**Nr.**	**Stachelhaus code**	**AA**
**Suomilide B-E, G & H**	*Nostoc* sp. KVJ20	BGC0003044	BslA	QQH17589.1	A1	**DAFFLGVTFK**	D-*allo*-Ile
**Suomilide A**	*Nodularia sphaerocarpa* UHCC 0038	BGC0002962	SuoB	ULP74384.1	A1	**DAFFLGVTFK**	D-*allo*-Ile
**Aeruginosin 98A-C**	*Microcystis aeruginosa* NIES-98	BGC0000298	AerB	ACM68684.1	A1	**DAFFLGVTFK**	D-*allo*-Ile
**Pseudospumigin A & E**	*Nostoc* sp. CENA543	BGC0001748	SpuB	ATP76246.1	A2	**DAFFLGVTFK**	L-Ile
**Ulleungmycin A & B**	*Streptomyces* sp. KCB13F003	BGC0001814	UlmA	ATU31794.1	A4	**DALWMGGVFK**	L-*allo*-Ile
**Desotamide A, C & D**	*Streptomyces scopuliridis* SCSIO ZJ46	Li et al.[9]	DsaH	AJW76710.1	A2	**DALWMGGVFK**	L-*allo*-Ile
**Varlaxin 1046A & 1022A**	*Nostoc* sp. UHCC 0870	Heinilä et al.[10]	VarB	UKO95748.1	A1	**DALWMGGVFK**	D-Ile

Table S4. Data points used for biphasic progress curve for suomilide B.

min[s]/c[nM]	2000	666.6667	222.2222	74.07407	24.69136	8.230453	2.743484	0.914495	0.304832	0.101611	0.03387	0.01129	0
**00:00**	3642*	3743*	3701*	3798*	4185	4406	4393	4618	4402	4289	4481*	4516*	4593
**00:45**	3686*	3721*	3670*	3828*	4327	4929	5073	5391	5310	5331	5597*	5689*	5318
**01:30**	3630*	3708*	3696*	3828*	4275	5377	5904	6187	5993	6102	6478*	6595*	6103
**02:15**	3630*	3742*	3663*	3806*	4231	5754	6428	6763	6790	6828	7098*	7064*	6819
**03:00**	3638*	3657*	5916*	3843*	4314	6010	7044	7442	7423	7481	7591*	7823*	7487
**03:45**	3630*	3657*	3716*	3834*	4274	6191	7616	8089	8156	8277	8413*	8571*	8324
**04:30**	3580*	3684*	3688*	3735*	4199	6373	8147	8695	8806	9037	9146*	9333*	9036
**05:15**	3581*	3679*	3712*	3770*	4199	6582	8755	9314	9550	9707	9748*	10122*	9711
**06:00**	3619*	3669*	3658*	3731*	4318	6659	9295	9946	10217	10410	10490*	10987*	10508
**06:45**	3641*	3637*	3628*	3705*	4353	6776	9621	10414	10844	11257	11367*	11736*	11190
**07:30**	3567*	3670*	3639*	3774*	4248	6759	10084	11067	11518	11971	11997*	12639*	11971
**08:15**	3611*	3651*	3600*	3742*	4298	6821	10637	11503	12075	12732	12815*	13447*	12726
**09:00**	3525*	3684*	3592*	3736*	4297	6833	11080	12135	12870	13342	13541*	14288*	13472
**09:45**	3504*	3618*	3627*	3749*	4305	6964	11359	12644	13485	14137	14194*	14957*	14294
**10:30**	3542*	3592*	3589*	3789*	4316	6992	11789	13110	14049	15040	14975*	15756*	14949
**11:15**	3498*	3632*	3593*	3704*	4249	7067	12164	13575	14589	15721	15642*	16525*	15692
**12:00**	3525*	3609*	3634*	3657*	4193	7160	12431	14016	15076	16234	16514*	17450*	16773
**12:45**	3532*	3599*	3594*	3802*	4248	7093	12849	14442	15838	17028	17046*	18245*	17228
**13:30**	3535*	3623*	3594*	3722*	4336	6987	13087	14957	16306	17656	17837*	18945*	17849
**14:15**	3520*	3646*	3589*	3751*	4166	7138	13613	15218	16948	18458	18473*	19593*	18727
**15:00**	3579*	3618*	3620*	3675*	4257	7182	13796	15675	17612	19322	19387*	20528*	19320
**15:45**	3563*	3655*	3576*	3681*	4240	7269	14067	15973	18146	19911	19973*	21237*	20302
**16:30**	3540*	3618*	3593*	3712*	4246	7218	14199	16329	18487	20650	20589*	21971*	20899
**17:15**	3615*	3637*	3580*	3652*	4264	7296	14506	16759	18916	21280	21422*	22859*	21614
**18:00**	3531*	3556*	3545*	3781*	4214	7311	14652	17123	19595	22054	22023*	23764*	22386
**18:45**	3493*	3655*	3604*	3664*	4241	7350	15084	17406	20277	22646	22795*	24267*	22998
**19:30**	3515*	3584*	3636*	3666*	4232	7359	15175	17895	20613	23223	23332*	24953*	23838
**20:15**	3519*	3616*	3565*	3756*	4232	7358	15549	18081	20991	23899	23890*	25722*	24681
**21:00**	3579*	3643*	3549*	3732*	4173	7356	15747	18587	21775	24902	24661*	26665*	25045
**21:45**	3469*	3631*	3553*	3696*	4263	7334	15776	18580	22163	25240	25263*	27491*	26013
**22:30**	3533*	3590*	3539*	3699*	4279	7378	15900	18901	22609	26032	25875*	28272*	26759
**23:15**	3450*	3621*	3555*	3743*	4229	7398	16209	19487	22906	26524	26492*	28684*	27389
**24:00**	3525*	3545*	3576*	3747*	4200	7506	16398	19789	23380	27318	27131*	29469*	28088
**24:45**	3551*	3592*	3542*	3657*	4283	7377	16597	19972	23775	28091	27858*	30276*	28833
**25:30**	3541*	3575*	3590*	3707*	4256	7456	16675	20263	24172	28651	28438*	30977*	29586
**26:15**	3489*	3592*	3539*	3644*	4247	7489	16877	20329	24612	29337	29312*	31775*	30240
**27:00**	3521*	3660*	3580*	3687*	4280	7501	17139	20640	24918	29864	29786*	32539*	30989
**27:45**	3494*	3602*	3560*	3705*	4243	7551	17151	20925	25556	30583	30137*	33204*	31427
**28:30**	3471*	3585*	3504*	3667*	4188	7490	17281	21131	25917	31223	30820*	33971*	32416
**29:15**	3542*	3556*	3619*	3648*	4259	7592	17612	21661	26365	31486	31641*	34735*	33080
**30:00**	3517*	3625*	3600*	3597*	4280	7569	17705	21758	26906	32417	32281*	35206*	33736
**30:45**	3554*	3612*	3587*	3725*	4202	7628	17783	21938	26841	32853	32943*	36183*	34827
**31:30**	3521*	3542*	3501*	3683*	4288	7567	17876	22214	27414	33702	33433*	36953*	35104
**32:15**	3567*	3617*	3475*	3637*	4251	7592	18088	22188	27833	34259	33809*	37193*	35620
**33:00**	3473*	3642*	3537*	3716*	4238	7603	18294	22626	28235	34976	34696*	38093*	36359
**33:45**	3505*	3561*	3570*	3721*	4193	7625	18403	22719	28368	35450	34965*	38796*	37102
**34:30**	3487*	3497*	3494*	3738*	4297	7763	18407	22783	29265	35864	35855*	39421*	37820
**35:15**	3558*	3566*	3572*	3708*	4267	7623	18424	23208	29224	36274	36381*	40073*	38282
**36:00**	3424*	3587*	3504*	3640*	4234	7716	18654	23343	29476	37299	36834*	40994*	39125
**36:45**	3564*	3569*	3530*	3650*	4260	7675	18658	23341	29922	37417	37209*	41698*	39483
**37:30**	3526*	3553*	3516*	3716*	4236	7732	18661	23635	30206	38217	37554*	42461*	40600
**38:15**	3501*	3519*	3536*	3679*	4269	7698	18982	23911	30583	38622	38618*	42919*	41435
**39:00**	3524*	3620*	3533*	3666*	4241	7820	18963	24200	31287	39144	39104*	43267*	41640
**39:45**	3473*	3599*	3531*	3652*	4243	7829	19289	24310	31410	39985	39453*	44236*	42373
**40:30**	3420*	3582*	3521*	3633*	4259	7832	19345	24560	31479	40385	40084*	44738*	43037
**41:15**	3483*	3534*	3522*	3715*	4309	7830	19413	24771	32244	41122	41085*	45578*	43799
**42:00**	3485*	3585*	3569*	3695*	4223	7856	19485	24927	32149	41682	41468*	46203*	44324
**42:45**	3489*	3541*	3559*	3684*	4290	7890	19476	24718	32974	41900	41898*	47073*	45359
**43:30**	3466*	3591*	3493*	3729*	4269	7839	19596	25077	33016	42610	42138*	47096*	45756
**44:15**	3468*	3539*	3575*	3675*	4249	7951	19656	25252	33140	43268	42734*	47880*	46200
**45:00**	3473*	3570*	3498*	3672*	4284	7972	19789	25466	33490	43599	43623*	48568*	46737

*Data points that were not considered for the generation of figure 5A.

Table S5. Data points used for IC_{50} curve for suomilide B, D, G, and H.

nM	Suomilide B			Suomilide D			Suomilide G			Suomilide H		
**2000**	0,074521	0,752997	3	-0,01544	0,061094	3	0,227066	0,993021	3	-0,09312	0,157933	3
**666,6667**	-0,22603	0,069401	3	0,074176	0,05198	3	-0,2436	0,121755	3	-0,12759	0,319259	3
**222,2222**	-2,1908	4,318921	3	0,747892	0,188073	3	-0,02855	0,344686	3	-0,09999	0,027102	3
**74,07407**	-0,40369	0,642819	3	3,434367	0,279854	3	0,021019	0,246813	3	0,082267	0,053609	3
**24,69136**	0,063086	0,10417	3	14,79376	1,276841	3	0,461707	0,039599	3	1,606472	0,240238	3
**8,230453**	3,468075	2,74251	3	53,70472	0,464334	3	2,23686	0,574319	3	6,537755	1,022546	3
**2,743484**	14,45921	1,708316	3	80,88529	0,797744	3	12,88524	2,750684	3	25,68718	1,49906	3
**0,914495**	18,9871	8,284493	3	91,97881	0,793734	3	35,86002	4,534692	3	67,30834	10,73139	3
**0,304832**	53,08255	9,053357	3	99,61673	2,628544	3	83,73835	2,895174	3	83,15048	4,44858	3
**0,101611**	76,49281	13,00042	3	97,06448	0,685342	3	94,11668	2,718206	3	95,42289	0,076698	3
**0,03387**	81,60782	3,43013	3	101,7913	3,823691	3	96,06712	0,800142	3	95,66421	3,808909	3
**0,01129**	98,01428	2,068298	3	101,4883	3,443525	3	102,9305	1,940885	3	101,0452	4,698513	3
**0**	100	0	3	100	0	3	100	0	3	100	0	3

REFERENCES

(1) Marty, M. T.; Baldwin, A. J.; Marklund, E. G.; Hochberg, G. K. A.; Benesch, J. L. P.; Robinson, C. V. Bayesian Deconvolution of Mass and Ion Mobility Spectra: From Binary Interactions to Polydisperse Ensembles. *Anal. Chem.* **2015**, 87 (8), 4370–4376. https://doi.org/10.1021/acs.analchem.5b00140.

(2) Dührkop, K.; Fleischauer, M.; Ludwig, M.; Aksenov, A. A.; Melnik, A. V.; Meusel, M.; Dorrestein, P. C.; Rousu, J.; Böcker, S. SIRIUS 4: A Rapid Tool for Turning Tandem Mass Spectra into Metabolite Structure Information. *Nat Methods* **2019**, 16 (4), 299–302. https://doi.org/10.1038/s41592-019-0344-8.

(3) Dührkop, K.; Nothias, L.-F.; Fleischauer, M.; Reher, R.; Ludwig, M.; Hoffmann, M. A.; Petras, D.; Gerwick, W. H.; Rousu, J.; Dorrestein, P. C.; Böcker, S. Systematic Classification of Unknown Metabolites Using High-Resolution Fragmentation Mass Spectra. *Nat Biotechnol* **2021**, 39 (4), 462–471. https://doi.org/10.1038/s41587-020-0740-8.

(4) Reher, R.; Kim, H. W.; Zhang, C.; Mao, H. H.; Wang, M.; Nothias, L.-F.; Caraballo-Rodriguez, A. M.; Glukhov, E.; Teke, B.; Leao, T.; Alexander, K. L.; Duggan, B. M.; Van Everbroeck, E. L.; Dorrestein, P. C.; Cottrell, G. W.; Gerwick, W. H. A Convolutional Neural Network-Based Approach for the Rapid Annotation of Molecularly Diverse Natural Products. *J. Am. Chem. Soc.* **2020**, *142* (9), 4114–4120. https://doi.org/10.1021/jacs.9b13786.

(5) Kim, H. W.; Zhang, C.; Reher, R.; Wang, M.; Alexander, K. L.; Nothias, L.-F.; Han, Y. K.; Shin, H.; Lee, K. Y.; Lee, K. H.; Kim, M. J.; Dorrestein, P. C.; Gerwick, W. H.; Cottrell, G. W. DeepSAT: Learning Molecular Structures from Nuclear Magnetic Resonance Data. *Journal of Cheminformatics* **2023**, *15* (1), 71. https://doi.org/10.1186/s13321-023-00738-4.

(6) Stachelhaus, T.; Mootz, H. D.; Marahiel, M. A. The Specificity-Conferring Code of Adenylation Domains in Nonribosomal Peptide Synthetases. *Chemistry & Biology* **1999**, *6* (8), 493–505. https://doi.org/10.1016/S1074-5521(99)80082-9.

(7) Zdouc, M. M.; Blin, K.; Louwen, N. L. L.; Navarro, J.; Loureiro, C.; Bader, C. D.; Bailey, C. B.; Barra, L.; Booth, T. J.; Bozhüyük, K. A. J.; Cediel-Becerra, J. D. D.; Charlop-Powers, Z.; Chevrette, M. G.; Chooi, Y. H.; D'Agostino, P. M.; de Rond, T.; Del Pup, E.; Duncan, K. R.; Gu, W.; Hanif, N.; Helfrich, E. J. N.; Jenner, M.; Katsuyama, Y.; Korenskaia, A.; Krug, D.; Libis, V.; Lund, G. A.; Mantri, S.; Morgan, K. D.; Owen, C.; Phan, C.-S.; Philmus, B.; Reitz, Z. L.; Robinson, S. L.; Singh, K. S.; Teufel, R.; Tong, Y.; Tugizimana, F.; Ulanova, D.; Winter, J. M.; Aguilar, C.; Akiyama, D. Y.; Al-Salihi, S. A. A.; Alanjary, M.; Alberti, F.; Aleti, G.; Alharthi, S. A.; Rojo, M. Y. A.; Arishi, A. A.; Augustijn, H. E.; Avalon, N. E.; Avelar-Rivas, J. A.; Axt, K. K.; Barbieri, H. B.; Barbosa, J. C. J.; Barboza Segato, L. G.; Barrett, S. E.; Baunach, M.; Beemelmanns, C.; Beqaj, D.; Berger, T.; Bernaldo-Agüero, J.; Bettenbühl, S. M.; Bielinski, V. A.; Biermann, F.; Borges, R. M.; Borriss, R.; Breitenbach, M.; Bretscher, K. M.; Brigham, M. W.; Buedenbender, L.; Bulcock, B. W.; Cano-Prieto, C.; Capela, J.; Carrion, V. J.; Carter, R. S.; Castelo-Branco, R.; Castro-Falcón, G.; Chagas, F. O.; Charria-Girón, E.; Chaudhri, A. A.; Chaudhry, V.; Choi, H.; Choi, Y.; Choupannejad, R.; Chromy, J.; Donahey, M. S. C.; Collemare, J.; Connolly, J. A.; Creamer, K. E.; Crüsemann, M.; Cruz, A. A.; Cumsille, A.; Dallery, J.-F.; Damas-Ramos, L. C.; Damiani, T.; de Kruijff, M.; Martín, B. D.; Sala, G. D.; Dillen, J.; Doering, D. T.; Dommaraju, S. R.; Durusu, S.; Egbert, S.; Ellerhorst, M.; Faussurier, B.; Fetter,

A.; Feuermann, M.; Fewer, D. P.; Foldi, J.; Frediansyah, A.; Garza, E. A.; Gavriilidou, A.; Gentile, A.; Gerke, J.; Gerstmans, H.; Gomez-Escribano, J. P.; González-Salazar, L. A.; Grayson, N. E.; Greco, C.; Gomez, J. E. G.; Guerra, S.; Flores, S. G.; Gurevich, A.; Gutiérrez-García, K.; Hart, L.; Haslinger, K.; He, B.; Hebra, T.; Hemmann, J. L.; Hindra, H.; Höing, L.; Holland, D. C.; Holme, J. E.; Horch, T.; Hrab, P.; Hu, J.; Huynh, T.-H.; Hwang, J.-Y.; Iacovelli, R.; Iftime, D.; Iorio, M.; Jayachandran, S.; Jeong, E.; Jing, J.; Jung, J. J.; Kakumu, Y.; Kalkreuter, E.; Kang, K. B.; Kang, S.; Kim, W.; Kim, G. J.; Kim, H.; Kim, H. U.; Klapper, M.; Koetsier, R. A.; Kollten, C.; Kovács, Á. T.; Kriukova, Y.; Kubach, N.; Kunjapur, A. M.; Kushnareva, A. K.; Kust, A.; Lamber, J.; Larralde, M.; Larsen, N. J.; Launay, A. P.; Le, N.-T.-H.; Lebeer, S.; Lee, B. T.; Lee, K.; Lev, K. L.; Li, S.-M.; Li, Y.-X.; Licona-Cassani, C.; Lien, A.; Liu, J.; Lopez, J. A. V.; Machushynets, N. V.; Macias, M. I.; Mahmud, T.; Maleckis, M.; Martinez-Martinez, A. M.; Mast, Y.; Maximo, M. F.; McBride, C. M.; McLellan, R. M.; Bhatt, K. M.; Melkonian, C.; Merrild, A.; Metsä-Ketelä, M.; Mitchell, D. A.; Müller, A. V.; Nguyen, G.-S.; Nguyen, H. T.; Niedermeyer, T. H. J.; O'Hare, J. H.; Ossowicki, A.; Ostash, B. O.; Otani, H.; Padva, L.; Paliyal, S.; Pan, X.; Panghal, M.; Parade, D. S.; Park, J.; Parra, J.; Rubio, M. P.; Pham, H. T.; Pidot, S. J.; Piel, J.; Pourmohsenin, B.; Rakhmanov, M.; Ramesh, S.; Rasmussen, M. H.; Rego, A.; Reher, R.; Rice, A. J.; Rigolet, A.; Romero-Otero, A.; Rosas-Becerra, L. R.; Rosiles, P. Y.; Rutz, A.; Ryu, B.; Sahadeo, L.-A.; Saldanha, M.; Salvi, L.; Sánchez-Carvajal, E.; Santos-Medellin, C.; Sbaraini, N.; Schoellhorn, S. M.; Schumm, C.; Sehnal, L.; Selem, N.; Shah, A. D.; Shishido, T. K.; Sieber, S.; Silviani, V.; Singh, G.; Singh, H.; Sokolova, N.; Sonnenschein, E. C.; Sosio, M.; Sowa, S. T.; Steffen, K.; Stegmann, E.; Streiff, A. B.; Strüder, A.; Surup, F.; Svenningsen, T.; Sweeney, D.; Szenei, J.; Tagirdzhanov, A.; Tan, B.; Tarnowski, M. J.; Terlouw, B. R.; Rey, T.; Thome, N. U.; Torres Ortega, L. R.; Tørring, T.; Trindade, M.; Truman, A. W.; Tvilum, M.; Udwary, D. W.; Ulbricht, C.; Vader, L.; van Wezel, G. P.; Walmsley, M.; Warnasinghe, R.; Weddeling, H. G.; Weir, A. N. M.; Williams, K.; Williams, S. E.; Witte, T. E.; Rocca, S. M. W.; Yamada, K.; Yang, D.; Yang, D.; Yu, J.; Zhou, Z.; Ziemert, N.; Zimmer, L.; Zimmermann, A.; Zimmermann, C.; van der Hooft, J. J. J.; Linington, R. G.; Weber, T.; Medema, M. H. MIBiG 4.0: Advancing Biosynthetic Gene Cluster Curation through Global Collaboration. *Nucleic Acids Res* **2025**, *53* (D1), D678–D690. https://doi.org/10.1093/nar/gkae1115.

(8) Terlouw, B. R.; Huang, C.; Meijer, D.; Cediel-Becerra, J. D. D.; Rothe, M. L.; Jenner, M.; Zhou, S.; Zhang, Y.; Fage, C. D.; Tsunematsu, Y.; Wezel, G. P. van; Robinson, S. L.; Alberti, F.; Alkhalaf, L. M.; Chevrette, M. G.; Challis, G. L.; Medema, M. H. PARAS: High-Accuracy Machine-Learning of Substrate Specificities in Nonribosomal Peptide Synthetases. *bioRxiv* January 10, **2025**, p 2025.01.08.631717. https://doi.org/10.1101/2025.01.08.631717.

(9) Li, Q.; Song, Y.; Qin, X.; Zhang, X.; Sun, A.; Ju, J. Identification of the Biosynthetic Gene Cluster for the Anti-Infective Desotamides and Production of a New Analogue in a Heterologous Host. *J. Nat. Prod.* **2015**, *78* (4), 944–948. https://doi.org/10.1021/acs.jnatprod.5b00009.

(10) Heinilä, L. M. P.; Jokela, J.; Ahmed, M. N.; Wahlsten, M.; Kumar, S.; Hrouzek, P.; Permi, P.; Koistinen, H.; Fewer, D. P.; Sivonen, K. Discovery of Varlaxins, New Aeruginosin-Type Inhibitors of Human Trypsins. *Org. Biomol. Chem.* **2022**, *20* (13), 2681–2692. https://doi.org/10.1039/D1OB02454J.
